# The British Society for Antimicrobial Chemotherapy Resistance Surveillance Project: methods and limitations

**DOI:** 10.1093/jac/dkaf248

**Published:** 2025-10-27

**Authors:** Michael Allen, Rosy Reynolds, Shazad Mushtaq, Olisaeloka Nsonwu, Russell Hope, Carolyne Horner, Christopher Longshaw, Benjamin J Parcell, David M Livermore

**Affiliations:** Medical Affairs, MSD (UK) Limited, 120 Moorgate, London EC2M 6UR, UK; British Society for Antimicrobial Chemotherapy, 53 Regent Place, Birmingham B1 3NJ, UK; British Society for Antimicrobial Chemotherapy, 53 Regent Place, Birmingham B1 3NJ, UK; Population Health Sciences, University of Bristol, Bristol BS8 2PS, UK; Antimicrobial Resistance and Healthcare Associated Infections Reference Unit, UK Health Security Agency, Colindale, London NW9 5EQ, UK; Antimicrobial Resistance and Healthcare Associated Infections Division, UK Health Security Agency, Colindale, London NW9 5EQ, UK; Antimicrobial Resistance and Healthcare Associated Infections Division, UK Health Security Agency, Colindale, London NW9 5EQ, UK; British Society for Antimicrobial Chemotherapy, 53 Regent Place, Birmingham B1 3NJ, UK; British Society for Antimicrobial Chemotherapy, 53 Regent Place, Birmingham B1 3NJ, UK; Scientific Affairs, Shionogi B.V., Fifty Paddington, 50 Eastbourne Terrace, Paddington W2 6LG, UK; Division of Population Health and Genomics, School of Medicine, University of Dundee, Ninewells Hospital and Medical School, Dundee DD1 9SY, UK; Department of Medical Microbiology, Ninewells Hospital and Medical School, Dundee DD1 9SY, UK; Antimicrobial Resistance and Healthcare Associated Infections Reference Unit, UK Health Security Agency, Colindale, London NW9 5EQ, UK; Norwich Medical School, University of East Anglia, Norwich NR4 7TJ, UK

## Abstract

**Objectives:**

The BSAC Bacteraemia and Respiratory Resistance Surveillance Programmes provided long-term surveillance of antibiotic resistance in key pathogens of bloodstream and both community- and hospital-acquired respiratory infections in the UK and Ireland. This paper details the methodologies used. Data limitations are discussed.

**Methods:**

Sentinel laboratories across the UK and Ireland contributed up to a fixed annual quota of isolates of defined bacterial groups. For each Programme, a Central Laboratory confirmed bacterial identifications, measured MICs by the BSAC agar dilution method, investigated mechanisms of resistance and determined serotypes of *Streptococcus pneumoniae*. Identification methods evolved over time, e.g. with adoption of MALDI-TOF. Classification of susceptibility and resistance follows the 2022 (not contemporaneous) EUCAST guidance.

**Results:**

Seventy-nine laboratories contributed 30 716 community respiratory isolates from 1999/2000 to 2018/19; 65 laboratories contributed 13 508 hospital respiratory isolates from 2008/09 to 2018/19; 81 laboratories contributed 56 064 bacteraemia isolates from 2001 to 2019. Although large and teaching hospitals were over-represented, the resistance rates for bacteraemia organisms collected in England mirror more extensive (but less standardized or detailed) national data gathered from laboratories by the UK Health Security Agency and its predecessor organizations, which provided a bespoke data extract.

**Conclusions:**

These surveillance Programmes have provided comprehensive and reliable information on antibiotic susceptibility in the UK and Ireland over two decades. Detailed results, showing resistance trends and mechanisms of antibiotic resistance, are presented in five papers in this Supplement.

## Introduction

The BSAC Resistance Surveillance Project, initiated in 1999, addressed concerns about rising antibiotic resistance and the paucity of longitudinal surveillance.^1–3^ It comprised Programmes for respiratory and bloodstream pathogens, generating quantitative susceptibility data and information regarding mechanisms of antibiotic resistance for the UK and Ireland, and was guided by the BSAC Working Party (later Standing Committee) on Resistance Surveillance.

The Respiratory Programme ran from October 1999 to September 2019. It initially focused on the three ‘typical’ bacterial agents of community-associated lower respiratory tract infections (CA-LRTI), namely, *Streptococcus pneumoniae*, *Haemophilus influenzae* and *Moraxella catarrhalis*, collecting these species during winter ‘seasons’ from October to April. From 2008/09 onwards, the scope widened to include the major pathogens of hospital-acquired lower respiratory tract infection (HA-LRTI), with the collection periods extended to full years, still starting each October. The Bacteraemia Programme commenced in 2001 and continued through 2019. It collected the pathogens found, by national public health surveillance, to cause most bacteraemias.

This paper describes the methods used to (i) collect isolates, (ii) confirm their identification and assess their antibiotic susceptibility, (iii) characterize resistance mechanisms or strain types, (iv) serotype *S. pneumoniae* and (v) analyse data. The challenges of undertaking the programmes and the limitations of the data are discussed. Full results are presented elsewhere in this Supplement.^4–8^ The isolates collected and data generated are now held as a bioresource for further research, available via the NHS Tayside Biorepository in Dundee.^9^

## Materials and methods

### Design

Both Programmes were sentinel surveillances. Selected collecting laboratories (Tables 1 and 2) sent isolates to a central laboratory for detailed testing. From 1999/2000 to 2012/13, the Central Laboratory for the Respiratory Programme was GR Micro in London (later Quotient Bioresearch Ltd, then LGC, Fordham, UK). From 2013/2014, coordination of this programme moved to the Health Protection Agency (later Public Health England, now UK Health Security Agency, UKHSA) Antimicrobial Resistance and Healthcare-Associated Infections Reference Unit (AMRHAI). AMRHAI was the central laboratory for the BSAC Bacteraemia Programme throughout its lifetime from 2001 to 2019.

**Table 1.** Numbers of collecting laboratories, isolate quotas and collection targets per species or organism collection group by year

(A) Community-associated LRTI					
Annual collection	Target	Each of *S. pneumoniae* and *H. influenzae*		*M. catarrhalis*	
Periods	*N* of centres	Quota/lab	Target	Quota/lab	Target
1999/00–2007/08^a^	20	50	1000	25	500
2008/09–2009/10^b^	20	25	500	13	260
2010/11–2014/15^b^	40	14	560	7	280
2015/16–2018/19^b^	25	20	500	10	250

(B) Hospital-acquired LRTI					
Annual collection	Target	Enterobacterales		Each of *S. aureus*, *Pseudomonas* spp. and *Acinetobacter* spp.	
Periods	*N* of centres	Quota/lab	Target	Quota/lab	Target
2008/09–2009/10^b^	20	50	1000	13	260
2010/11–2014/15^b^	40	28	1120	7	280
2015/16–2018/19^b^	25	40	1000	10	250

(C) Bacteraemia					
Annual collection periods	Target	Each of *E. coli* and *S. aureus*		Each other collection group^d^	
	*N* of centres	Quota/lab	Target	Quota/lab	Target
2001–07^c^	25	10	250	10	250
2008–09^c^	25	20	500	10	250
2010–15^c^	40	14	560	7	280
2016–19^c^	25	20	500	10	250

^a^Collection periods, October–April.

^b^Collection periods, October–September.

^c^Calendar years, January–December.

^d^Bacteraemia surveillance included 11 separate collection groups other than *E. coli* and *S. aureus*: *Klebsiella*, *Enterobacter*, Proteeae, *Pseudomonas*, other Gram-negative bacteria (2001–07), *Serratia* (2008–19), CoNS, *Enterococcus*, *S. pneumoniae*, other α-haemolytic streptococci and β-haemolytic streptococci. (*Serratia* were collected as part of the mixed ‘other Gram-negative bacteria’ group until 2007 and as a single genus from 2008).

**Table 2.** Numbers of laboratories that contributed isolates, by country

Annual collection	Target	Number of laboratories contributing (range)				
Period	*N* of centres	Ireland	N. Ireland	Scotland	Wales	England
CA-LRTI						
1999/00–2009/10	20	2–3	1–2	2–4	2	12–13
2010/11–2014/15	40	4	1	2–4	1–3	25–30
2015/16–2018/19	25	2	2	2	1	17–18
HA-LRTI						
2008/09–2009/10	20	3	1	4	2	11–12
2010/11–2014/15	40	4	1	2–4	1–3	24–30
2015/16–2018/19	25	2	2	2	1	17
Bacteraemia						
2001–09	25	2	1–2	2	2	17
2010–15	40	4	1–3	2–3	1–3	25–31
2016–19	25	2	2	2	1	17–18

Collecting laboratories were selected by the Central Laboratory for each Programme to give good geographical coverage of the UK and Ireland, with a range of catchments (urban/rural, teaching/non-teaching hospitals, more/less socially deprived). If a laboratory withdrew at the end of a collection period, it was either replaced by a laboratory nearby or serving a similar population. If a site withdrew mid-period, or failed to collect isolates, the Programme ran with fewer sites, with a replacement sought for the next collection year.

Funding was raised by the BSAC from pharmaceutical company sponsors (see Results and discussion and Acknowledgements). The Society called for tenders for the execution of each Programme at initiation, with a re-tendering in 2013. Central Laboratories were appointed based upon detailed project plans and costings, as submitted to the Society. These plans were then further developed and reviewed annually by the BSAC, which funded a part-time surveillance co-ordinator throughout.

### Collection of isolates

The target numbers of collecting laboratories, quotas and total collection targets per season/year by period and organism collection group for the Bacteraemia and Respiratory Programmes are detailed in Table 1. Initially, both Programmes used 20–25 sentinel laboratories separately selected by the two central laboratories, with only partial overlap between the collecting laboratories contributing to each of the two Programmes. From 2009/10 (Respiratory) and 2010 (Bacteraemia), the collecting laboratories were increased to 40 and, from 2013/14 and 2014, were standardized across both Programmes. Finally, from 2015 to 2016 (Respiratory) and 2016 (Bacteraemia), the number of collecting laboratories was reduced to 25, partly owing to financial pressure and partly because laboratory mergers meant that many sites were collecting isolates from multiple hospital trusts. The distribution and number of collecting laboratories by country and by collection period are detailed for both Programmes in Table 2.

Isolates were collected as consecutive, clinically significant and non-duplicate. Clinical significance was adjudged by a clinical microbiologist at the collecting laboratory (i.e. present in such numbers, or numbers of blood culture bottles, to indicate infection). Duplicate isolates were defined as of the same species, from the same patient and body site, within 7 days or, from 2006/07 (Respiratory) and 2008 (Bacteraemia), 14 days.

#### BSAC Respiratory Programme

From 1999/00 to 2018/19, the Respiratory Programme collected ‘typical’ CA-LRTI species (i.e. *S. pneumoniae*, *H. influenzae* and *M. catarrhalis*) from patients with confirmed or presumed LRTI in community settings or hospitalised for ≤48 h. The collection period ran for a ‘winter season’ between 1 October and 30 April until 2007/08. From 2008/09, this was extended to 1 October until 30 September, reflecting the addition of HA-LRTI, which is less seasonal. The HA-LRTI component collected defined numbers of isolates per season for *S. aureus*, *P. aeruginosa*, *Acinetobacter* spp. and Enterobacterales, then known as Enterobacteriaceae^10^ (Table 1) from patients with confirmed or presumed LRTI who had been hospitalized for >48 h. Isolates from patients living with cystic fibrosis were excluded from both the CA-LRTI and HA-LRTI surveillances.

#### BSAC Bacteraemia Programme

The BSAC Bacteraemia surveillance initially sought 250 isolates per species group annually from 25 laboratories, rising to 280 isolates from 40 laboratories from 2010 to 2015 inclusive (Table 1). For *E. coli* and *S. aureus*, the target totals were doubled from 2008. From 2016 to 2019, the number of collecting laboratories was reduced to 25. A mixed ‘other Gram-negative’ group was included until 2007 but was dominated by *Serratia* spp. and was replaced with a specific *Serratia* group thereafter. Isolates of *K. aerogenes* were known and collected as *Enterobacter aerogenes* (i.e. within *Enterobacter* quotas) until 2018 but thereafter in the *Klebsiella* quota, reflecting revised taxonomy.^11^ To assess the representativeness of the collected data for bacteraemia, a comparison was undertaken using a bespoke extract generated by the UKHSA retrospectively (after the BSAC Bacteraemia Programme closed), from their voluntary bacteraemia surveillance for England.^12^ The UKHSA has no parallel scheme for LRTI, so similar validation could not be undertaken.

### Patient characteristics

Only limited anonymized information was collected for patient context at the time of sampling, including age, sex, care setting, hospital speciality and probable source of infection. Details are provided in Table 3.

**Table 3.** Patient context at time of sample

(A) Community-associated lower respiratory infection 1999/00–2018/19			
Information	Seasons	Categories	% complete^a^
Age	All	Years; grouped for analysis	>99%
Sex	All	Male/female	>99%
Care setting^b^	All	Community settings^c^ Hospital settings (≤48 h since admission)^c^	>97%^d^
Specimen type	All	E.g. sputum and bronchoalveolar lavage	>98%^e^
ICU speciality	2013/14–2018/19	Intensive/critical care (ICU)—yes/no	76%–90%^f^

(B) Hospital-acquired lower respiratory infection 2008/09–2018/19			
Information	Seasons	Categories	% complete^g^
Age	All	Years; grouped for analysis	>99%
Sex	All	Male/female	>99%
Care setting^h^	All	Hospital >48 h since admission	>99%
Specimen type	All	E.g. sputum and bronchoalveolar lavage	>99%
Hospital speciality	2008/09–2014/15	Accident and emergency; cardiovascular; care of the elderly; high dependency (HDU); general medicine; haematology/oncology; intensive/critical care; nephrology; paediatrics; surgery; ‘other’	96%–98%^i^
ICU speciality^j^	All	Intensive/critical care (ICU)—yes/no	94%–99%^i^

(C) Bacteraemia 2001–19			
Information	Years	Categories	% complete^k^
Age	All	Years (grouped for analysis)	>98%
Sex	All	Male/female	>99%
Care setting	2002–19^l^	Community and outpatient settings Hospital ≤48 h since admission Hospital >48 h since admission	>97%
Hospital speciality^m^	2003–13	Accident and emergency; cardiovascular; care of the elderly; general medicine; haematology/oncology; intensive/critical care; nephrology; paediatrics; surgery; ‘other’	96%–99%^n^
ICU speciality^o,p^	2003–19	Intensive/critical care (ICU)—yes/no	91%–99%^p^
Probable source of infection^q^	2001–13	Genitourinary system (including urinary catheters); lines and devices (excluding urinary catheters); respiratory tract; gastrointestinal/intra-abdominal; skin and soft tissue (including wounds but not surgical site wounds); endocarditis; surgical site wounds; cerebrospinal fluid; ‘other’	53%–74%^r^

^a^Percentages calculated annually, for all CA-LRTI isolates combined.

^b^The CA-LRTI protocol excluded samples taken >48 h since hospital admission but occasional (<0.1%) non-compliant isolates remain in dataset (2014 and later).

^c^The CA-LRTI surveillance counted hospital outpatients as ‘hospital’ until 2012/13 and as ‘community’ from 2013/14.

^d^97.8% in 2002/03 due to one centre confirming only that its 47 isolates were compliant (i.e. not hospital >48 h after admission); otherwise >99%.

^e^Mean 99.9%, after excluding 1999/00 when one centre confirmed only that its 91 isolates were from lower respiratory sources.

^f^Completeness among isolates from hospital settings only.

^g^Percentages calculated annually, for all HA-LRTI isolates combined.

^h^The HA-LRTI protocol excluded samples taken ≤48 h since hospital admission or in community settings but very occasional (<0.1%) non-compliant isolates remain in dataset (2015/16 and later).

^i^Mean 97%.

^j^Intensive/critical care units; does not include other high-dependency units or beds.

^k^Percentages calculated annually, for all bacteraemia isolates combined. Data completeness for some variables, particularly speciality and focus of infection, varied by organism group.

^l^Care setting was also recorded in 2001, but only 84% complete.

^m^Data for speciality/ICU from 2001 to 2002 are excluded as discrepant; data collection forms were improved from 2003 onwards. Hospital speciality was recorded in nine defined categories (plus ‘other’) until 2013. Free text entries were accepted in addition in 2014–15 but were too disparate for meaningful analysis apart from ICU/non-ICU.

^n^Overall, 2% missing and 6% recorded as ‘other’.

^o^From 2016, speciality categories were simplified to intensive/critical care units (ICUs) and all other units (including community and outpatient settings).

^p^Completeness for ICU speciality remained ≥96% until 2015, before declining to reach 91% in 2019.

^q^Recorded in eight defined categories (plus ‘other’) until 2013. The inclusion of free text entries in 2014–15 did not increase data completeness or intelligibility. Data not sought after 2015.

^r^Overall, 38% missing and 3% ‘other’. Of those with data, 88% were in the five categories first named.

### Storage and transport of isolates

Whilst the Respiratory Programme was run by GR Micro and its successors, there was a requirement for isolates to be stored frozen using a bead/cryovial system (Pro-Lab Diagnostics, Wirral, UK), or similar. For the Bacteraemia Programme throughout and the Respiratory Programme from 2013/14 onwards, collecting laboratories stored isolates using local methods in suitable media at or below −70°C for up to 12 months or at −20°C for shorter periods compatible with very high rates of recovery (≤2 months for *S. pneumoniae* and *H. influenzae*).

Before transport to a Central Laboratory, thawed isolates were sub-cultured to non-selective media to give luxuriant overnight growth. This was transferred to agar slopes or suspended in a transport medium and sent to the relevant Central Laboratory in compliance with prevailing transport regulations.^13,14^ At the Central Laboratory, isolates were stored at or below −70°C in blood glycerol broth, or by other agreed established methods giving a high long-term recovery (e.g. storage on beads at −70°C).

### Microbiological methods

#### Bacterial identification

All microbiological testing was performed centrally. From 1999/00 to 2012/13, respiratory isolates were tested by GR Micro and their successors. Subsequently, until the Project's end, respiratory isolates were tested at the AMRHAI. Bacteraemia isolates were tested at the AMRHAI throughout.

On receipt at the Central Testing Laboratory, isolates were sub-cultured to non-selective media and checked for purity. Those not meeting inclusion/exclusion criteria were discarded, with replacements sought. The methodology for identification (Table 4) evolved during the Project's lifespan. Initially, it primarily involved chromogenic media, API strips and PCR for species identification within CoNS and enterococci.^16–18^ MALDI-TOF (Bruker Biotyper, Bruker, Bremen, Germany) was introduced in 2011/12 as an acceptable alternative identification method in the Respiratory Programme, for all organisms except *S. pneumoniae*, and became standard for most species from 2013/14 in the Respiratory Programme and from 2014 in the Bacteraemia Programme.

**Table 4.** Methods for microbial identification by surveillance Programme, organism group and period

Organism/group	Period	Method
(A) Respiratory Programme		
CA-LRTI		
*S. pneumoniae*	1999/00–2012/13	Gram-positive diplococci, growing as α-haemolytic sometimes umbonate or mucoid colonies on horse blood agar. Catalase negative with a positive optochin test and/or bile solubility test
	2013/14–2018/19	Optochin sensitivity and bile solubility
*H. influenzae*	1999/00–2010/11	Gram-negative coccobacilli, requiring both X (haematin) and V (NAD) factor to grow on a non-supplemented medium
	2011/12–2012/13	As above, or MALDI-TOF (Bruker Daltonics, Bremen Germany)
	2013/14–2018/19	MALDI-TOF (Bruker)
*M. catarrhalis*	1999/00–2010/11	Gram-negative diplococci, producing whitish/grey colonies on horse blood or chocolate horse blood agar. Oxidase positive, butyrate esterase positive
	2011/12–2012/13	As above, or MALDI-TOF (Bruker)
	2013/14–2018/19	MALDI-TOF (Bruker)
HA-LRTI		
*E. coli*	2008/09–2010/11	API20E (bioMérieux, Basingstoke, UK)
	2011/12–2012/13	As above, or MALDI-TOF
	2013/14–2018/19	Pink growth on CHROMagar™ Orientation (CHROMagar, Paris, France) and/or Brilliance UTI Clarity Agar (Oxoid/Thermo Fisher Scientific). Alternatively, MALDI-TOF (Bruker)
Enterobacterales^a^ other than *E. coli*	2008/09–2010/11	API20E (bioMérieux)
	2011/12–2012/13	As above, or MALDI-TOF
	2013/14–2018/19	MALDI-TOF (Bruker)
*Pseudomonas*	2008/09–2010/11	API20NE (bioMérieux)
	2011/12–2012/13	API20NE (bioMérieux), alternatively, MALDI-TOF (Bruker)
	2013/14–2018/19	MALDI-TOF (Bruker)
*Acinetobacter*	2008/09–2010/11	API20NE (bioMérieux)
	2011/12–2012/13	API20NE (bioMérieux); alternatively, MALDI-TOF (Bruker)
	2013/14–2016/17	PCR for *bla*_OXA-51_ to identify *A. baumannii.*^15^ If negative, then MALDI-TOF (Bruker) or *rpoB* sequencing^16^
	2017/18–2018/19	PCR for *bla*_OXA-51_ to identify *A. baumannii.*^15^ If negative, then MALDI-TOF (Bruker)
*S. aureus*	2008/09–2010/11	White or yellow colonies on horse blood or chocolate horse blood agar. Catalase positive, positive agglutination with staphylococcal latex, (Oxoid/Thermo Fisher Scientific, Basingstoke, UK). DNase production
	2011/12–2012/13	As above. Alternatively, MALDI-TOF (Bruker)
	2013/14–2018/19	MALDI-TOF (Bruker) in conjunction with CHROMagar™ *Staphylococcus*/chromogenic media (BioConnections, Knypersley, UK). Alternatively, coagulase tests
(B) Bacteraemia Programme		
Gram-positive BSI		
*S. aureus*	2001–11	Coagulase test and chromogenic media (Oxoid/Thermo Fisher, Basingstoke, UK)
	2012–13	As above. Alternatively, MALDI-TOF (Bruker)
	2014–19	MALDI-TOF (Bruker) in conjunction with CHROMagar™ *Staphylococcus* (BioConnections, Knypersley, UK); alternatively, coagulase tests
CoNS*^b,c^*	2001–05	Coagulase test and PCR to identify species^17^
	2006–12	Coagulase test, CHROMagar™ *Staphylococcus*/chromogenic media
	2013–19	MALDI-TOF (Bruker), to identify species, in conjunction with *Staphylococcal* CHROMagar (manufactured by CHROMagar, distributed by BioConnections, Knypersley, UK)/chromogenic media and coagulase tests
Enterococci*^d^*	2001–11	PCR for *ddI* and biochemical tests^18^
	2012–13	PCR for *ddI* and biochemical tests.^18^ Alternatively, MALDI-TOF
	2014–19	MALDI-TOF
*S. pneumoniae*	2001–13	Optochin sensitivity, bile solubility
	2014–16	Optochin sensitivity, bile solubility if optochin-resistant
	2017–19	WGS if isolate also received by UKHSA reference service Otherwise, as above
*α*- and non-Haemolytic streptococci	2001–16	ID32 STREP kits (bioMérieux) and additional biochemical tests for definitive identification by the Respiratory and Vaccine Preventable Bacteria Reference Unit (RVPBRU)^19^
	2017–19	MALDI-TOF (Bruker), identifying to species-group level (e.g. ‘*mitis* group’)
*β*-Haemolytic streptococci	2001–13	Lancefield grouping (Prolex Streptococcal Grouping Latex Kits, Pro-Lab Diagnostics, Merseyside, UK)
	2014–19	MALDI-TOF (Bruker). If Lancefield group could not be inferred from the species identification (e.g. *Streptococcus dysgalactiae*), then Lancefield typing (Prolex Streptococcal Grouping Latex Kits, Pro-Lab Diagnostics)
Gram-negative BSI		
*E. coli*	2001–02	API20E (bioMérieux)
	2003–11	Pink growth on CHROMagar™ Orientation and/or Brilliance™, UTI Clarity™ Agar (Oxoid/Thermo Fisher Scientific. Questionable results retested with API20E (bioMérieux)
	2012–19	Pink growth on CHROMagar™ Orientation and/or Brilliance UTI Clarity™ Agar (Oxoid/Thermo Fisher Scientific. Alternatively, MALDI-TOF (Bruker)
Enterobacterales other than *E. coli*	2001–11	API20E (bioMérieux)
	2012–13	API20E (bioMérieux). Alternatively, MALDI-TOF (Bruker)
	2014–19	MALDI-TOF (Bruker)
*Pseudomonas* ^e^	2001–02	API20NE (bioMérieux)
	2003–11	Blue/green colour on *Pseudomonas* P/King's A medium (Oxoid/Thermo Fisher) to confirm *P. aeruginosa*. Others tested with API 20NE (bioMérieux)
	2012	Blue/green colour on *Pseudomonas* P/King's A medium (Oxoid/Thermo Fisher) to confirm *P. aeruginosa*. Alternatively, MALDI-TOF (Bruker)
	2013–19	MALDI-TOF (Bruker)

MALDI-TOF was introduced in 2011/12 as an acceptable alternative identification method in the Respiratory Programme for all organisms except *S. pneumoniae* and became standard for most from 2013/14.

^a^The HA-LRTI surveillance collected isolates in the order Enterobacterales under the former family name Enterobacteriaceae throughout. The membership of the family Enterobacteriaceae was narrowed when the order Enterobacterales was published in 2016, but collecting laboratories continued to supply the full former range of organisms including, e.g. *Serratia* and members of the former tribe Proteeae (now classified, respectively, in families Yersiniaceae and Morganellaceae within Enterobacterales).

^b^CoNS were identified to species level 2001–05 and 2013–19, but not 2006–12.

^c^Primer pairs for the identification of CoNS species were adapted from Gribaldo et al.^17^

^d^Primer pairs for the identification of the enterococcal species were adapted from Dutka-Malen et al.^18^

^e^Any non-fermenters collected as ‘other Gram-negative bacteria’ (2001–07) were tested in the same way as *Pseudomonas*.

Isolates that gave doubtful results or had anomalous antibiograms for their species were re-identified by a second method, generally with API20E or API20NE strips (bioMérieux, Basingstoke, UK) for Gram-negative bacteria, PCR for enterococci and Lancefield typing for β-haemolytic streptococci. Taxonomic changes were retrospectively applied, with isolates assigned according to the taxonomy current in March 2023, as detailed in the *List of Prokaryotic names with Standing in Nomenclature*.^20^

#### Discrepant identifications, over-quota isolates and mixed cultures

When collecting and Central Laboratory identifications differed, the isolate remained eligible for inclusion under its central laboratory identification. Replacements for ineligible isolates were sought, up to the quota, if time remained in the collecting period. If a collecting laboratory submitted more than its quota of isolates, excess isolates were excluded starting with any submitted under other names and then by the most recent isolate. In cases of mild contamination, attempts were made to purify the primary organism. Grossly mixed cultures were discarded; if time remained in the collecting season, a replacement was sought.

#### Susceptibility testing

MICs were determined by BSAC agar dilution, as summarized in Table S1 (available as Supplementary data at *JAC* Online). This method remained essentially unchanged throughout the Project.^21^ Special conditions applied for a few sponsored antibiotics: in particular, Ca^2+^-supplemented isotonic agar (Mast Group Ltd, Bootle, UK) was used for daptomycin. Agar plates of 10 × 10 cm (10 × 10 inoculation spots) were used by GR Micro and its successors whereas 8.5 × 12.8 cm plates (8 × 12 spots) were used by AMRHAI. These contained 50 or 40 mL of agar, respectively.

MICs were read manually at GR Micro and its successors. At AMRHAI, MICs were read using the Sorcerer Image Analysis System, as periodically updated (Perceptive Instruments Ltd, Haverhill, UK), with visual confirmation where required. The density of bacterial suspensions was checked by dilution and spiral plating to ensure the correct inoculum of 10^4^ cfu/spot or, for *M. catarrhalis* with β-lactams, 10^6^ cfu/spot. The ‘other Gram-negative’ collection (2001–07) in the Bacteraemia Programme included occasional anaerobes and Category 3 pathogens; these were tested using Etests (AB Biodisk, Solna, Sweden; later bioMérieux, Basingstoke, UK). Handling of any Category 3 pathogens received in the ‘other Gram-negative’ or ‘Enterobacterales’ groups followed the prevailing health and safety guidance of the UKHSA and its predecessor organizations.

Table 5 lists antibiotics that were tested for at least 2 years and considered by the BSAC to be of continuing clinical or surveillance interest, in some cases for distinguishing resistance types; other agents tested are listed in footnotes. The selection of agents tested each year evolved over time as clinical practice changed or sponsored antibiotics lost exclusivity. Antibiotics were sourced from Sigma-Aldrich (Poole, UK) or, if sponsored, from their manufacturers (see Funding). Antibiotic dilution ranges aimed to give full endpoints and avoid off-scale values (recorded as MIC ≤ X mg/L or >X mg/L) so far as possible.

**Table 5.** Antibiotics of continued clinical or surveillance interest tested in the BSAC Programmes and duration of testing

	Number of years/seasons tested											
	Lower respiratory tract infections							Bacteraemia				
	Community			Hospital				Gram +			Gram −	
Antibiotic	*S. pneumoniae*	*H. influenzae*	*M. catarrhalis* ^a^	*S. aureus*	Enterobacterales	*Pseudomonas*	*Acinetobacter*	Staphylococci	Enterococci	Streptococci	Enterobacterales	*Pseudomonas*
Amikacin^b^	x	x	x	4	4	4	4	4	x	x	4	4
Amoxicillin^c^	20	20	3	x	11	x	x	x	x	19	19	x
Ampicillin^c^	x	16	4	x	x	x	x	x	19	x	x	x
Carbenicillin^c,d,e^	x	x	x	x	x	6	x	x	x	x	x	6
Cefaclor^c^	6	6	3	x	x	x	x	x	x	x	x	x
Cefotaxime^c^	20	20	8	x	11	x	x	x	x	19	18	x
Cefoxitin^c,d^	x	x	x	x	11	x	x	x	x	x	19	x
Ceftaroline^b^	3	3	3	3	x	x	x	5	2	5	3	x
Ceftazidime^c^	x	x	x	x	11	11	11	x	x	x	19	19
Ceftazidime/avibactam^b^	x	x	x	x	3	3	3	x	x	x	4	4
Ceftobiprole^b^	8	8	8	8	8	8	8	16	15	15	15	15
Ceftolozane/tazobactam^b^	2	2	2	2	9	9	9	x	x	7	9	9
Cefuroxime^c,e^	15	15	12	x	x	x	x	x	x	x	13	x
Ciprofloxacin^c^	20	20	18	11	11	11	11	19	15	15	19	19
Clarithromycin^b^	4	4	3	x	x	x	x	x	x	x	x	x
Clindamycin^c^	20	x	x	11	x	x	x	19	x	19	x	x
Co-amoxiclav^c^	x	20	18	11	11	x	x	x	x	x	19	x
Colistin^c^	x	x	x	x	9	9	9	x	x	x	9	9
Daptomycin^b,f^	x	x	x	x	x	x	x	7	7	6	x	x
Ertapenem^b^	x	x	x	x	5	x	x	2	6	11	11	x
Erythromycin^c^	20	16	18	11	x	x	x	19	15	19	x	x
Fusidic acid^c^	x	x	x	11	x	x	x	17	x	x	x	x
Gentamicin^c^	x	x	x	11	11	11	11	19	19	19	19	19
Imipenem^b,c^	x	x	x	x	10	10	10	2	13	19	18	18
Imipenem/relebactam^b^	x	x	x	x	5	5	5	x	x	5	5	5
Levofloxacin^b,g^	3	3	3	x	x	x	x	x	x	x	x	x
Linezolid^b^	x	x	x	6	x	x	x	14	14	14	x	x
Meropenem^b,c^	x	x	x	x	7	7	7	x	3	9	10	10
Minocycline^b,c^	9	9	9	7	5	x	5	14	12	12	12	x
Moxifloxacin^b,g^	6	6	4	x	x	x	x	x	x	x	x	x
Mupirocin^c^	x	x	x	11	x	x	x	13	x	x	x	x
Nalidixic acid^h^	x	3	3	x	x	x	x	x	x	x	x	x
Oxacillin^c^	x	x	x	11	x	x	x	19	x	15	x	x
Penicillin^c^	20	x	x	7	x	x	x	15	15	19	x	x
Piperacillin/tazobactam^b,c^	x	x	x	5	11	11	11	12	13	13	19	19
Rifampicin^c^	x	x	x	11	x	x	x	17	x	x	x	x
Streptomycin^c^	x	x	x	x	x	x	x	x	4	x	x	x
Tedizolid^b^	x	x	x	5	x	x	x	5	5	5	x	x
Teicoplanin^c^	x	x	x	11	x	x	x	19	19	19	x	x
Tetracycline^c^	20	20	18	11	7	x	9	19	14	19	14	x
Tigecycline^c^	9	9	9	5	5	x	5	12	12	12	12	x
Tobramycin^c^	x	x	x	x	6	6	6	x	x	x	6	6
Trimethoprim^c^	16	16	4	11	5	x	x	19	x	x	5	x
Vancomycin^c^	x	x	x	11	x	x	x	19	19	19	x	x
Maximum years possible	20	20	18	11	11	11	11	19	19	19	19	19

Agents tested for a single year are excluded. Exclusions apply where an agent lacks activity against some species within a group; e.g., colistin was tested for 9 years against bloodstream *E. coli*, *Klebsiella* and *Enterobacter* spp. but not against Proteeae or *Serratia* spp.Data are also available for the following agents, but are of limited value because (i) the agents have been abandoned from development or withdrawn from the UK or (ii) were tested only for a single year (number of years tested): azithromycin (1), BAL30072 (1), BAL30072/meropenem (1), delafloxacin (1), doripenem (7), faropenem (3), gemifloxacin (4), solithromycin (1) and telavancin (7).The following agents were tested for short periods, and the results on file remain confidential to the sponsoring company or its successor: cethromycin (ABT773, 1), ceftaroline/avibactam (1), lefamulin (1), razupenem (1), cefilavancin (TD1792, 1) and telithromycin (3).The following agents were tested singly in some years to support interpretation of results for their typical use in combination: avibactam, ceftolozane, clavulanic acid, relebactam and tazobactam.The following agents were tested under non-standard conditions in 1 or 2 years for comparison: cefoxitin at 30°C, ceftobiprole in Columbia/salt agar, oxacillin with 48 h of incubation.Results for the following agents are not available: dalbavancin (2 years, invalid to test on agar).x: not tested or tested in only 1 year.

^a^MICs were not measured for *M. catarrhalis* in 2001/02 or 2003/04.

^b^Sponsored by a pharmaceutical company in at least 1 year.

^c^Included in the BSAC protocol without sponsorship in at least 1 year.

^d^Tested as an aid to interpretive reading of resistance mechanisms.

^e^Also tested against *selected* isolates of relevant organisms in further years to aid interpretive reading.

^f^Daptomycin was also tested for 1 year in the Respiratory Programme.

^g^Levofloxacin and moxifloxacin were also tested for single years in the Bacteraemia Programme.

^h^Nalidixic acid was used to screen for reduced susceptibility to quinolones.

All MICs reported in this Supplement were reviewed against EUCAST v12.0 (2022) criteria.^22^ Details of the breakpoints used are provided in the Supplementary data for the five publications of the substantive results in this Supplement.^4–8^

#### Quality assurance of MIC testing

For each collection period, the Central Laboratory measured MICs for internal quality control strains,^21^ tested in parallel with the collected isolates. Susceptibility results for test isolates were included in the analysis if the results for these controls fell within the range accepted at the time. If a run was rejected based on the failed internal controls, testing was repeated for the rejected antibiotic(s) only.

#### Detection of mechanisms of resistance and additional typing

##### β-Lactamases in fastidious Gram negatives

All *H. influenzae* and *M. catarrhalis* isolates were tested for β-lactamase with nitrocefin (Becton Dickinson, Wokingham, UK).

##### ESBLs, AmpC and K1 enzymes

Methods for the detection of ESBLs and AmpC enzymes in Enterobacterales were gradually refined but, in general, were applied to all isolates with ceftazidime or cefotaxime MICs ≥ 1 mg/L or, pre-2007, with ceftazidime ≥2 mg/L.

ESBL activity was sought by determining MICs of ceftazidime, cefotaxime and cefepime each ±4 mg/L clavulanate, using BSAC agar dilution or Etests. Cefpirome was used as a substitute for cefepime when cefepime was unavailable. Swarming Enterobacterales (i.e. *Proteus* spp.) were always tested by Etest. ESBL production was inferred when any (but generally all) of the cephalosporin MICs were reduced ≥8-fold (i.e. ≥3 doubling dilutions) by clavulanate. An exception was made for *Klebsiella oxytoca* isolates considered to be K1 hyperproducers based on the criteria below, as these can give weak false positive results in clavulanate synergy testing with cefotaxime, cefepime or cefpirome (but not ceftazidime).^23^

AmpC activity was inferred by (i) testing cefotaxime ±100 mg/L cloxacillin by BSAC agar dilution or (ii) based upon cefotetan resistance, as tested by BSAC agar dilution or with Etests. Copious AmpC production was inferred when the cefotaxime MIC was reduced ≥4-fold (i.e. by ≥2 doubling dilutions) by cloxacillin, so long as this interpretation was compatible with the rest of the antibiogram (i.e. relatively susceptible to cefepime and cefpirome but resistant to ceftazidime, cefotaxime and piperacillin/tazobactam, though with AmpC-derepressed *Serratia* remaining susceptible to ceftazidime and AmpC-derepressed *Morganella morganii* to piperacillin/tazobactam).^23^

Isolates of *K. oxytoca* with piperacillin/tazobactam MICs ≥ 128 mg/L were additionally tested, by BSAC agar dilution, with aztreonam and cefuroxime. High-level resistance to these agents, but not to ceftazidime, together with borderline resistance to cefotaxime, was taken to indicate hyperproduction of K1 β-lactamase.^23^

From 2003, Enterobacterales inferred to have ESBLs based on cephalosporin–clavulanate synergy were subjected to type-specific PCR for *bla*_CTX-M_.^24^ A cefotaxime MIC higher than the ceftazidime MIC was also required for confirmation that only a CTX-M ESBL was present; PCR for *bla*_VEB_ and *bla*_PER_ was performed for highly ceftazidime-resistant *P. aeruginosa* (MIC > 64 mg/L) lacking carbapenemases.^25,26^ From 2013 (Bacteraemia) and 2013/14 (LRTI), *E. coli*, *Klebsiella* and *Proteus mirabilis* inferred to have AmpC-mediated resistance, based upon cefotaxime/cloxacillin synergy, were subjected to PCR for plasmid-mediated AmpC.^27^ Similar tests were not performed on *Enterobacter*, *Morganella*, *Providencia* and *Serratia* spp., because AmpC phenotypes in these species largely reflect mutational derepression of endogenous chromosomal β-lactamases.

##### Carbapenemase

From the 2013/14 (Respiratory) and 2014 (Bacteraemia) collections onwards, all carbapenem-non-susceptible Enterobacterales, except Proteeae with inherent borderline resistance to imipenem (MICs 2–4 mg/L), were examined further. Methods were refined over time, notably with the inclusion of a ‘phenotypic testing’ MIC run. This included MIC determinations for imipenem ±320 mg/L EDTA (on Mueller–Hinton agar, not Iso-Sensitest), temocillin and aztreonam, as well as the cephalosporin/clavulanate and cefotaxime/cloxacillin combinations used to detect ESBL and AmpC activity (above). Metallo-β-lactamase (MBL) production was suspected if the isolate was resistant to cephalosporins, without clavulanate or cloxacillin synergy, but showed ≥8-fold imipenem/EDTA synergy. A Class A carbapenemase (e.g. KPC) was suspected if the isolate was resistant to carbapenems, but the temocillin MIC remained ≤32 mg/L, with no imipenem/EDTA synergy but with (in years where these were tested) imipenem/relebactam and ceftazidime/avibactam synergy. Possible OXA-48-like activity was suspected if the isolate was resistant to at least one carbapenem and lacked imipenem/EDTA synergy, and the temocillin MIC was ≥128 mg/L. These tests guided the use of specific single PCRs to seek carbapenemase genes.^28^ From 2017, a multiplex PCR (AusDiagnostics, Mascot, Australia) was used to seek, in parallel, genes for VIM, IMP, SME, OXA-48-like, KPC, NDM, SIM, FRI, IMI, SPM and GES carbapenemases in isolates flagged by phenotypic testing as possible carbapenemase producers.^29^

*Pseudomonas* spp. with antibiograms compatible with carbapenemase production (imipenem/meropenem MIC > 16 mg/L and ceftazidime MIC > 64 mg/L) were examined for carbapenemase genes by specific PCR, as for Enterobacterales.^28,30^ All isolates of *Acinetobacter* spp., regardless of antibiogram, were examined with a multiplex PCR that sought *bla*_OXA-51_, as a marker for *Acinetobacter baumannii*, along with the prevalent acquired carbapenemases of the genus: *bla*_OXA-23_, *bla*  _OXA-24/40_, *bla*  _OXA-58_ and *bla*  _OXA-143_.^15^

##### Methicillin and mupirocin resistance among staphylococci

PCR testing for *mecA* was introduced for all *S. aureus* from 2005 and for all CoNS from 2006.^31,32^ Thereafter, staphylococci were considered ‘methicillin-resistant’ if they were *mecA*-positive irrespective of MIC data; previously categorization was based upon oxacillin MICs. From 2006, *mupA*, encoding high-level mupirocin resistance, was also sought in all staphylococci by PCR.^33^

#### Serotyping of S. pneumoniae

Serotypes of bloodstream *S. pneumoniae* were determined throughout; those of CA-LRTI pneumococci were determined in the 2005/06 season and continuously from 2013/14 onwards. Methods evolved over time. From 2001 to 2004, bloodstream pneumococci were typed by slide agglutination, using latex antisera (Pneumotest-Latex Kit) and pneumococcal antisera (both from Statens Serum Institute, Copenhagen, Denmark). Subsequently, from 2005, bloodstream isolates were screened with a multiplex ELISA, using Luminex xMAP technology (Bioplex System, Bio-Rad, Hemel Hempstead, UK), with slide agglutination tests if a serotype was not identified.

Alternatively, from 2017, serotypes for most (*c.* 70%) bloodstream pneumococci were inferred from WGS data, based on the fact that many of these same BSAC-collected isolates (as tracked by sending Laboratory Reference Numbers) were also received and sequenced under ongoing UKHSA surveillance of invasive pneumococcal disease.^34^ For this WGS, pneumococci were grown on horse blood agar (UKHSA Media Services) and lysed using the Qiagen-recommended method for Gram-positive bacteria (Qiagen, Manchester, UK). Genomic DNA was extracted with a QIAsymphony SP automated instrument (Qiagen) and QIAsymphony DSP DNA Mini Kit, following the protocol for Gram-negative bacteria. DNA concentrations were measured using the Quant-IT Broad Range DNA Kit (Life Technologies, Paisley, UK) and GloMax 96 Microplate Luminometer (Promega, Southampton, UK). After adjusting to the required concentration, DNA was sent for WGS by Illumina methodology, using a whole-genome kmer comparison to confirm the species and the PneumoCaT tool to predict the serotype.^35^ For bacteraemia isolates collected by the BSAC but not received under UKHSA surveillance (∼30%), serotyping continued by classical methodology, as above.

In 2005/06, the serotypes of respiratory *S. pneumoniae* were identified by a two-stage process: first to serogroup level using the Pneumotest-Latex Kit and then to serotype level by Quellung reaction, using pneumococcal capsular antisera (kit and antisera from Statens Serum Institute, Copenhagen, Denmark). Isolates that gave no capsular reaction were sent to the Statens Serum Institute for further examination. From 2013/14 to 2018/19, respiratory *S. pneumoniae* were serotyped as per post-2005 bacteraemia isolates; WGS was not performed.

### Analysis

Analysis was descriptive and largely graphical, using Stata 18.0 (StataCorp LLC: College Station, TX, USA) and Bischoff's colour vision-sensitive ‘plotplainblind’ graph scheme.^36^ Missing data were excluded in the calculation of percentages. Missing data for patient characteristics, and details of a few exclusions from the BSAC MIC results owing to data anomalies, are noted in the Supplementary data of the related papers in this Supplement describing the results of the surveillance.^4–8^

Serotype diversity, excluding untyped isolates, was described by the Gini–Simpson diversity index (1 minus Simpson's sum of squared probabilities),^37^ calculated in Stata using the entropyetc package (N.J. Cox, 2024, http://fmwww.bc.edu/RePEc/bocode/e/).

## Results and discussion

A total of 79 laboratories contributed 30 716 isolates from CA-LRTI from 1999/00 to 2018/19; 65 sites contributed 13 508 HA-LRTI isolates from 2008/09 to 2018/19; and 81 collecting laboratories contributed 56 064 isolates to the BSAC Bacteraemia Programme over the 19 years (2001–19). Site turnover for both programmes, LRTI and BSI, averaged two changes (range 0–5) each year for collections targeting 20–25 collecting laboratories and six (range 4–8) for those targeting 40, but 12 changes when the Central Laboratory changed in 2013/14.

The substantive results of the surveillance are described in five publications in this Supplement.^4–8^ The present discussion confines itself to the merits, challenges and limitations of the Project as a whole over its two-decade lifespan.

The advantages of centralized testing, compared with compilation of hospitals’ routine susceptibility testing data (as in the UKHSA bacteraemia surveillances and EARS-net),^2,38,39^ include (i) greater granularity, with MICs rather than simple susceptibility categorization, (ii) standardized testing with a consistent core panel of antibiotics, (iii) the ability to investigate unusual isolates in detail and (iv) the possibility of testing new and developmental agents. Limitations are discussed below under the headings of Scale and representativeness, Laboratory methods and data quality, Antibiotics tested and Resistance mechanisms and strain types.

### Scale and representativeness

The main organism groups in the BSAC Bacteraemia Programme represented a very high proportion of the increasing number of bacteraemias (excluding those due to *Treponema*) reported to UKHSA by NHS-related laboratories in England (85% of approximately 59 200 in 2001, falling to 80% of 174 400 in 2019 and 80% of 215 200 in 2024, Figure S1). However, the number of isolates sampled and tested (2520–3377 annually, from 20–40 laboratories) was necessarily small compared with the national totals.^2,39^ This limitation of size reduced the power to detect subtle trends, led to more year-to-year ‘noise’ in resistance time–trend plots and made it extremely difficult to obtain a statistically representative sample of clinical isolates. The typical target sample was 250 isolates per organism group per year, but numbers were smaller for, e.g. species subgroups. The resulting variability in annual estimates of resistance prevalence is illustrated by simulation in Table S2 and Figures S2–S9. Other factors such as outbreaks, differences in resistance by collecting centre, centre turnover and experimental (laboratory) variation over time will all add to the variability.

Other limitations of scale and representativeness are subtler, but important. First, an issue arises because some species are easier to collect than others. In the Bacteraemia Programme, for example, target numbers of *E. coli* and *S. aureus* were reached early each year, with none collected later, meaning that any seasonal fluctuation in strain types and resistance would not be represented. Second, at its outset, the Project sought to include a representative mixture of institution types but gradually came to be dominated by tertiary hospital laboratories as these were the most reliable collectors. This bias was partly balanced by these sites coming to serve, and provide isolates from, increasing numbers of smaller peripheral hospitals. Third, whilst the inclusion of two or three centres from each of Wales, Scotland, the Republic of Ireland and Northern Ireland over-represented these polities on a population basis, it was clearly insufficient to give a comprehensive picture of their resistance trends. EARS-net data suggest several differences in resistance trends between the UK and Ireland, based upon wider sampling in the latter country. For example, the fall in MRSA rates has been slower and less complete in Ireland (38%–43% of all bloodstream *S. aureus* between 2000 and 2005 and 12%–18% between 2015 and 2019) than in the UK (corresponding ranges 41%–47% versus 6%–11%).^38^ Similarly, there were too few centres per region within England for statistically robust inter-region comparison, though there are and have been periods when resistance—as with ESBL *E. coli*—has been geographically clustered.^40,41^

For the Bacteraemia Programme, it was possible to compare BSAC results with collated national data for England, provided by the UKHSA as a bespoke and comprehensive data extract from the CDR module (formerly CoSurv/LabBase2) of their Second-Generation Surveillance System (SGSS) system.^2^  ^,39^ This scheme has collected diagnostic laboratories’ voluntarily submitted data for bacteraemia since the late 1980s, recording around 215 000 episodes in 2024. Its comprehensiveness has increased over time, with almost all NHS laboratories in England now participating; its data quality has also improved, reflecting wide adoption of MALDI-TOF for identification and better standardized (BSAC and EUCAST) susceptibility testing. Comparison to mandatory *S. aureus*/MRSA and *E. coli* bacteraemia surveillance suggests that, for these species, the proportions of isolates ‘captured’ rose from 82% in 2011 to 93% in 2019 for *S. aureus* and from 85% (2012) to 92% (2019) for *E. coli.*^40,42^ These comparisons are extremely useful. Where the very different BSAC and UKHSA surveillances identify the same trend among bloodstream isolates (e.g. declining MRSA,^5^ or rising, then stabilizing, ESBL *E. coli*,^6^ confidence in the trend is increased.

The extract of national bacteraemia data was for England only, with no similar comparison available for Wales, Scotland, Northern Ireland or the Republic of Ireland. Moreover, there is no equivalent UKHSA routine data collection for respiratory isolates, and this lack of large-scale comparator is a limitation of the BSAC Respiratory Programme.

Limitations of the UKHSA bacteraemia database—despite its value as a comparator—include the following: (i) not all relevant antibiotics are routinely tested at all laboratories; (ii) the UKHSA dataset is poorly representative for recently licensed and second-line agents, though there have been substantial increases in routine testing of linezolid and daptomycin over time,^43^ (ii) some species identifications in the UKHSA data are suspect, particularly before the wide adoption of MALDI-TOF, with significant likely error rates (e.g. ampicillin-resistant enterococci reported as *Enterococcus faecalis*); and (iii) the UKHSA data are scored against contemporaneous, not current, susceptibility criteria, as used here for the BSAC data.^2^  ^,39^

### Laboratory methods and data quality

The adoption of MALDI-TOF in the latter half of the BSAC Project's surveillance period provided more precise identification of collected isolates. It does however beg the question of how many earlier isolates might have been reassigned (principally to minor species) had they been subjected to this method.

Changes in taxonomy presented a further challenge, in particular, with the shift of *E. aerogenes* to the genus *Klebsiella*, as *K. aerogenes.*^10^ This directly impacted the denominator for the Bacteraemia Programme. Instead of ∼200 *Enterobacter* with ∼30 *E. aerogenes* per annum, its analysis includes fewer *Enterobacter* (principally *E. cloacae* group) and excess *Klebsiella*, making the collections more mismatched than intended. The Respiratory Programme was unaffected because Enterobacterales were collected as a single group.

BSAC agar dilution on Iso-Sensitest agar, as employed by the surveillance and summarized in Table S1, is an established technique but is decreasingly used nationally or internationally. It differs from the broth microdilution method advocated by CLSI and, subsequently, by EUCAST^44^ in two important respects. First, Iso-Sensitest agar is less rich than Mueller–Hinton media, reducing bacterial growth. Second, a single pre-existing mutant cell may grow to give turbidity in broth, whereas it would yield a single, discounted colony in agar dilution. Changing to broth microdilution was repeatedly debated by the BSAC Working Party but was rejected as disruptive to data continuity.

Next, we chose to apply EUCAST version 12.0 breakpoints (January 2022),^22^ retrospectively, to all BSAC data rather than using contemporaneous breakpoints. This was clearly preferable to having different breakpoints for the same antibiotic in different periods but has several consequences. First, as already noted, it creates complexity in comparing BSAC results to UKHSA data. Second, although EUCAST aims to ensure that their breakpoints do not split a susceptible wild-type population, problems arise if raised MICs for a substantial proportion of isolates are adjacent to the breakpoint, especially if this is then changed. Throughout much of the testing period, whilst the piperacillin/tazobactam breakpoint for Enterobacterales was 16 + 4 mg/L, we recorded significant numbers of isolates with MICs of exactly that value. These scored as susceptible and, since control MICs were ‘in range’ and resistance rates did not look unusual, no repeat testing was undertaken. Subsequently, the piperacillin/tazobactam breakpoint was lowered to 8 + 4 mg/L (albeit, latterly, with 16 + 4 mg/L as an ‘Area of Technical Uncertainty’), and these isolates moved to resistant, causing ‘spikes’ of resistance that would have been investigated, with retesting, had they been apparent at the time of the original testing. In retrospect, more weight should have been placed on control MICs being randomly scattered within acceptable ranges and clustered around their modes—as EUCAST now specifies—rather than simply being within range.^22^

In the first six collection periods for the Project, an analysis of repeated MIC measurements showed reasonable reproducibility within each central laboratory, with 50% of repeated MIC measurements agreeing exactly, 90% within ±1 dilution and 98% within +2 dilutions.^45^ Nevertheless, over 40% of repeated MIC measurements did not agree exactly, and this degree of experimental variation can be important, for example, if it substantially arises *between* experimental runs, rather than among isolates *within* a run. Typically, for annual collections of 200–250 isolates, a whole season's collection of a particular species were tested in only two or three runs, each of *c.* 90 isolates, over a short period. In these circumstances, between-run variation could translate into experimental variation between seasons, increasing noise and, where MICs were close to breakpoint, giving spurious resistance spikes.^46^ This issue was exemplified for MRSA collected in the Bacteraemia Programme between 2001 and 2007.^47^ Longitudinal data suggested slight year-on-year upward creep for glycopeptide MICs.^48^ However, re-tests, using the same central laboratory but with isolates from different years mixed between runs, demonstrated much less variability and failed to confirm the rising MICs over time.^48^

More generally, testing drug combinations, specifically β-lactams/β-lactamase inhibitor combinations, is technically more difficult than testing single agents. It is telling that they account for most of the instances where, for Enterobacterales, EUCAST allows an ‘Area of Technical Uncertainty.^22^ For co-amoxiclav, there is the further complication that EUCAST, and the BSAC Project, moved from testing with a 2:1 ratio to testing with a fixed 2 mg/L clavulanate.^49^ This creates a data discontinuity, for there is no way to review MICs obtained by the ratio method against breakpoints predicated upon a fixed clavulanate concentration.

### Antibiotics tested

Next, it was not possible to maintain a continuous testing record for all the clinically important antibiotics selected as representatives. Some were only developed during the surveillance period, and inclusion of those in pre-authorization development or patent-protected exclusivity depended on sponsorship by their manufacturers, which could be intermittent (e.g. tigecycline). An antibiotic might drop out through lack of sponsorship, particularly after losing exclusivity, but be included again later owing to its growing clinical importance (e.g. daptomycin). The resulting gaps detract from longitudinal analysis. Other changes over time in the antibiotic testing panel complicated interpretation of mechanisms. For example, imipenem was tested as the representative carbapenem until meropenem lost exclusivity. Thereafter, meropenem, as the most used analogue, replaced imipenem. This created an issue for *P. aeruginosa*, where resistance to meropenem, involving both OprD loss and efflux, is more complex than for imipenem, which most often solely involves loss of OprD.^50^

### Resistance mechanisms and strain types

The identification and characterization of resistance mechanisms developed over time, with tests added, e.g. as CTX-M ESBLs and carbapenemases, gained importance.^51^ Initially, the process of identifying mechanisms was *ad hoc* but was increasingly formalized in the annual project protocols. In compiling this Supplement, we ran additional molecular tests on a few early isolates that were unusually resistant but had no mechanisms contemporaneously recorded. This included oxacillin-resistant *S. aureus* with no *mecA* recorded and carbapenem-resistant Enterobacterales with no carbapenemase gene sought. Constraints of funding precluded wide-ranging investigations of resistance mechanisms; the Project prioritized those perceived to be of greatest public health concern. Had it begun now, two decades later, much wider use would have been made of WGS, which has become progressively less expensive and more available.

Serotyping of *S. pneumoniae* was undertaken throughout the Bacteraemia Programme but, for the Respiratory Programme, was performed only for one early season (2005/06) and then consistently from 2013 to 2014 season. This precluded precise analysis of the early impact of conjugate vaccine deployment in CA-LRTI, during a period when there were dramatic serotype shifts in bacteraemia.^4^ Except for pneumococci, little typing was done, even though it is well known that (i) much of the MRSA problem in the UK reflected the dissemination of just two lineages, EMRSA-15 and EMRSA-16;^52^ (ii) much of the UK's problem with ESBL-producing *E. coli* reflects dissemination of ST131 variants;^51^ (iii) much of the carbapenem resistance in *A. baumannii* is (or was) linked with two lineages, OXA-23 Clones 1 and 2,^53^ that spread among hospitals; and (iv) much of the high-level gentamicin resistance in *E. faecalis* in the early part of the present century was associated with two strains, both also highly resistant to fluoroquinolones.^54^

A final limitation is that data collection and testing for this surveillance ended 6 years ago and that the subsequent period, until this Supplement, included the major hiatus of the COVID-19 pandemic. During the lockdowns and associated restrictions, there was a remarkable suppression of *S. pneumoniae* bacteraemias (and, putatively, pneumonias).^55^ There also was a *c.* 13% fall in the number of *E. coli* bacteraemias,^56^ perhaps because the patients who would have contracted these instead succumbed to COVID-19 or because those with bacteraemias succumbed without presenting for care.

## Conclusions

The two BSAC Resistance Surveillance Programmes provided consistent and reliable information on antibiotic susceptibility in the UK and Ireland by using an adequate number of contributing centres and isolates, combined with standardized microbiological methods applied by a Central Laboratory, and suitable methods of statistical analysis. The information is more detailed than that available from routine data collections because MICs, not just S/I/R categories, are recorded for every organism and agent. The results from the Project are presented in five papers in this Supplement.^3–8^ The study database and isolate collections are now curated by Ninewells Hospital, Dundee, and the University of St Andrews and are available for further academic research, as outlined in the ‘Legacy’ paper in this Supplement.^9^

## Supplementary Material

dkaf248_Supplementary_Data

## Transparency declarations

M.A. is a Trustee of the BSAC Council and is employed by MSD (UK) Limited, London, UK. M.A. has shareholdings in Merck & Co., Inc., Rahway, NJ, USA. S.M., O.N. and R.H. are employees of the UKHSA. R.H. and O.N. work for the Antimicrobial Resistance and Healthcare Associated Infections Division and have no conflict of interest to declare. S.M. works for the Antimicrobial Resistance and Healthcare Associated Infections Reference Unit (AMRHAI), which has received financial support for conference attendance, research projects or contracted evaluations from numerous sources, including Accelerate Diagnostics, Allecra Therapeutics, AstraZeneca UK Ltd, AusDiagnostics, Basilea Pharmaceutica, Becton Dickinson Diagnostics, bioMérieux, Bio-Rad Laboratories, BSAC, Cepheid, Check-Points B.V., UK Department of Health, Enigma Diagnostics, Food Standards Agency, GlaxoSmithKline Services Ltd, Helperby Therapeutics, Henry Stewart Talks, IHMA Ltd, Innovate UK, Integra holdings, MSD, Meiji Seika Pharma Co. Ltd, Mobidiag, Momentum Biosciences Ltd, Neem Biotech, Nordic Pharma Ltd, Norgine Pharmaceuticals, Paratek Pharmaceuticals, Roche, Smith & Nephew UK Ltd, Shionogi & Co. Ltd, Trius Therapeutics, VenatoRx Pharmaceuticals and Wockhardt Ltd. C.H. was employed by Una Health Limited and QuantuMDx Group Limited during the period of data analysis and manuscript preparation. C.L. is a Trustee of the BSAC Council and is employed by Shionogi B.V. B.J.P.: consultancy: GenPax. D.M.L.: advisory boards or *ad hoc* consultancy: AdjuTec, AstraZeneca, Centauri, GenPax, GSK, Lipovation, Meiji, Paion, Shionogi, Sumitovant, Thermo Fisher and Wockhardt; paid lectures: bioMérieux, Pfizer, Shionogi and Zuellig Pharma; and relevant shareholdings or options: GenPax, GSK, Merck & Co Inc and Revvity amounting to less than 10% of portfolio value. D.M.L. also has shareholdings in Arecor, Genedrive, GENinCode, Probiotix, Rua Life Sciences, SkinBioTherapeutics and Verici Dx (all with research/products pertinent to medicines or diagnostics) through Enterprise Investment Schemes, but has no authority to trade these holdings directly. All other authors have nothing to declare.

## Supplementary data

Figures S1–S9 and Tables S1 and S2 are available as Supplementary data at *JAC*  Online.
