# Supplementary material for: The British Society for Antimicrobial Chemotherapy Resistance Surveillance Project: methods and limitations
Source: J Antimicrob Chemother. 2025 Oct 27;80(Suppl 4):iv7–iv21. doi: 10.1093/jac/dkaf248 (PMC12555735; doi:10.1093/jac/dkaf248)
Supplement: dkaf248_Supplementary_Data [file dkaf248_supplementary_data.pdf]

The British Society for Antimicrobial Chemotherapy Resistance Surveillance Project: methods and limitations SUPPLEMENTARY INFORMATION

Contents

UKHSA recording of bacteraemia episodes in England 2

Figure S1. Yearly number of bacteraemias reported to UKHSA by NHS-related laboratories in England, by organism (inclusion/non-inclusion in BSAC bacteraemia surveillance). 2

MIC testing in the BSAC Resistance Surveillance Project 3

Table S1. Summary of MIC testing method – conditions and media 3

Variation in estimates of resistance prevalence, illustrated by simulation 4

Caveat 4

Method 4

Notes 4

Table S2. 5th/95th and 25th/75th centiles of simulated estimates of resistance prevalence by sample size and true resistance rate 5

Figure S2. 20% resistance: centiles of simulated estimates for samples of [A] 20–900 isolates; [B] 100–500 isolates 6

Figure S3. 10% resistance: centiles of simulated estimates for samples of [A] 20–900 isolates; [B] 100–500 isolates 7

Figure S4. 5% resistance: centiles of simulated estimates for samples of [A] 20–900 isolates; [B] 100–500 isolates 8

Figure S5. 2% resistance: centiles of simulated estimates for samples of [A] 20–900 isolates; [B] 100–500 isolates 9

Impact of variation in 20-year surveillance series, illustrated by simulation 10

Figure S6. Six random examples of variation over simulated 20-year series for stable 10% resistance, sample size 200 10

Figure S7. Comparison by sample size: six superimposed simulated examples of 20-year series for stable 10% resistance 11

Figure S8. Comparison by true resistance rate: six superimposed simulated examples of 20-year series with 200 isolates/year 11

Visual impact of graph scale in 20-year surveillance series 12

Figure S9. Four simulated examples of 20-year series for stable 10% resistance presented on two scales: y-axis 0–20% or 0–50% 12

UKHSA recording of bacteraemia episodes in England

UKHSA and its predecessors have collected diagnostic laboratories' voluntarily-submitted routine data for bacteraemia since the late 1980s. The data represented in Figure S1 below were extracted from the CDR module (formerly CoSurv/LabBase2) of its Second-Generation Surveillance System (SGSS) system.

Records of *Treponema* and *Helicobacter* detected by blood tests were excluded from the extract; the same approach is taken by ESPAUR (English Surveillance Programme for Antimicrobial Utilisation and Resistance).

Reports of the same species (or, for streptococci, sub-species) were excluded as duplicates using a static 14-day window, retaining the most resistant as the reported sensitivity result.

Figure S1 categorises UKHSA-reported bacteraemias as due to "BSAC" or "Other" organisms. "BSAC organisms" are those in genera or tribes intentionally collected in all 19 years of the BSAC Bacteraemia Resistance Surveillance Programme and analysed in this JAC supplement i.e. (Gram-positive) *Staphylococcus*, *Streptococcus* and *Enterococcus*, and (Gram-negative) *Escherichia coli, Enterobacter, Klebsiella, Serratia*, Proteeae and *Pseudomonas*.

The many genera collected in small numbers as "other Gram-negative bacteria" in 2001–07 are not counted with BSAC organisms for this purpose.

Figures similar to S1 showing the numbers in each of the BSAC organism collection groups are in the Supplementary information of the two bacteraemia papers in this supplement.

Figure S1. Yearly number of bacteraemias reported to UKHSA by NHS-related laboratories in England, by organism (inclusion/non-inclusion in BSAC bacteraemia surveillance).

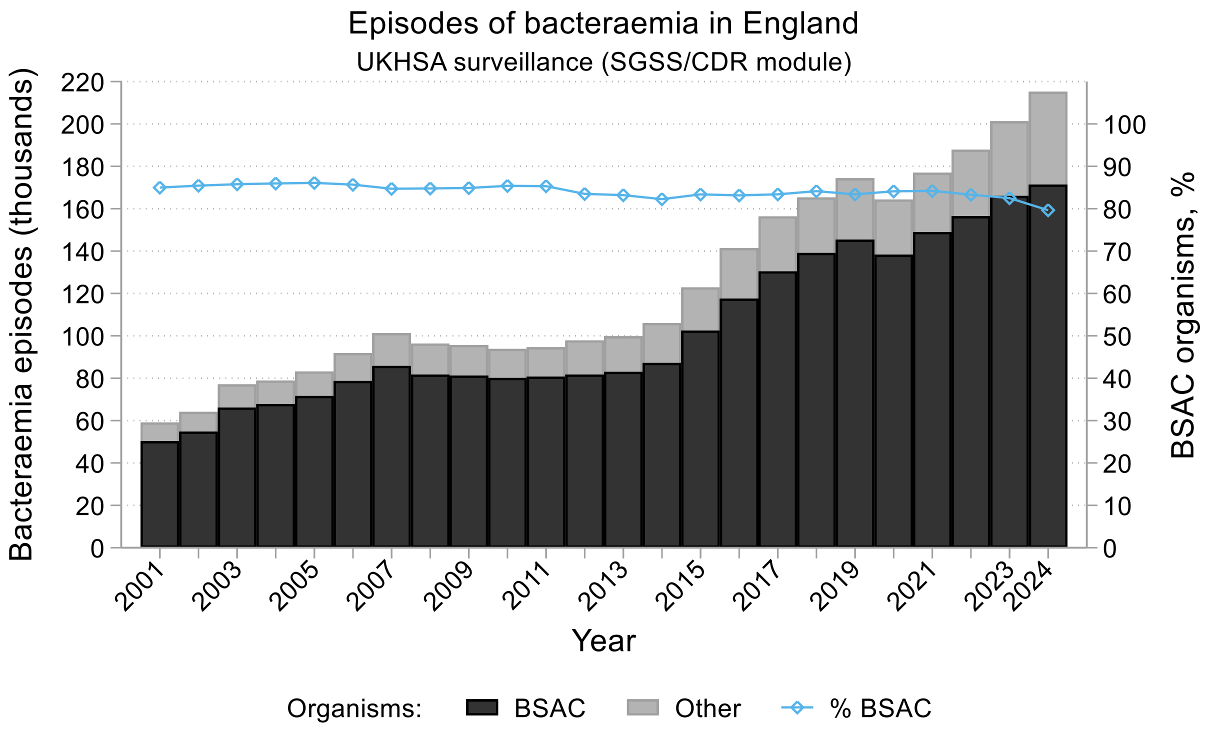

BSAC organisms: (Gram-positive) *Staphylococcus*, *Streptococcus*, *Enterococcus*; (Gram-negative) *Escherichia, Enterobacter, Klebsiella, Serratia*, Proteeae, *Pseudomonas*.
Note: *Treponema* (and *Helicobacter* detected by antibody tests) are excluded from UKHSA totals.

MIC testing in the BSAC Resistance Surveillance Project

Table S1. Summary of MIC testing method – conditions and media

Organism	Medium	Supplements	Spot size (CFU/spot)	Atmosphere	Temperature & duration
S. pneumoniae and other α- & non-haemolytic streptococci	IsoSensitest agar	5% defibrinated horse blood	10^4	air plus 4–6% CO_2	35–37°C 18–20h
β-haemolytic streptococci	IsoSensitest agar	5% defibrinated horse blood	10^4	air	35–37°C 18–20h
Staphylococci (**tests other than oxacillin**)	IsoSensitest agar	None	10^4	air	35–37°C 18–20h
Staphylococci (**oxacillin**)	Columbia agar	2% NaCl	10^4	air	30°C 24h
Enterobacterales (excluding swarming species)	IsoSensitest agar	None	10^4	air	35–37°C 18–20h
Swarming Enterobacterales e.g. *Proteus* spp.	IsoSensitest agar	50 mg/L PNPG[1]	10^4	air	35–37°C 18–20h
Acinetobacter spp.	IsoSensitest agar	None	10^4	air	35–37°C 18–20h
Pseudomonas spp.	IsoSensitest agar	None	10^4	air	35–37°C 18–20h
Haemophilus influenzae	IsoSensitest agar	5% defibrinated horse blood + 20 mg/L NAD	10^4	air plus 4–6% CO2	35–37°C 18–20h
Moraxella catarrhalis	IsoSensitest agar	5% defibrinated horse blood	10^4 (10^6 against β-lactams)	air	35–37°C 18–20h

[1] 1-(4-nitrophenyl)glycerol

Variation in estimates of resistance prevalence, illustrated by simulation

Caveat

These illustrations show only the variation due to statistical sampling. This is the minimum amount of variation possible for an ideal survey, assuming that resistance is stable over time and is detected with complete consistency, all isolates have the same probability of being resistant, and are independent of each other, and the intended sample size is always achieved exactly.

In reality, many other factors such, as outbreaks, differences in resistance by centre, centre turnover and experimental (laboratory) variation over time will all add to the variability.

Method

We considered four 'true' stable resistance rates (2%, 5%, 10% and 20%) and sample sizes between 20 and 900 isolates for illustration. We simulated 100,000 sample estimates of resistance prevalence for each sample size and 'true' resistance rate using the r(binomial) function in Stata 18.0 (StataCorp, College Station, TX) to give 100,000 simulated sample estimates of resistance prevalence. [1]

The distribution of these 100,000 sample estimates is discrete, not continuous, because an estimate of resistance can only take values corresponding to whole numbers of resistant isolates in the sample. It is summarised in Table S2 and Figures S1–S4, below, by its 5th, 25th, 75th and 95th centiles. At least 50% of all estimates must lie on or between the 25th and 75th centiles, and at least 90% on or between the 5th and 95th. Correspondingly, the proportions falling below the 5th or above the 95th centile can, collectively, be up to 10%.

Notes

The saw-tooth appearance of the graphs of centiles against sample size is a well-known corollary of the discrete nature of the binomial distribution.[2] It is particularly pronounced for small samples, in which a single isolate accounts for a relatively large fraction of the total sample, and there are correspondingly large gaps between the possible values of the estimated proportion resistant.

It is also well-known that the range between corresponding percentiles (such as 5th and 95th) of the sample estimate is not necessarily symmetrical about the true value. This affects estimates of low resistance rates the most, partly for the obvious reason that the lowest percentiles cannot be below zero while there is no corresponding constraint at the higher end. It also affects small samples more than large ones. Note that Table S2 understates the issue, because it happens to include favourable combinations of resistance rates and sample sizes: the graphs show how the 'saw teeth' interact to create asymmetry at other sample sizes.

In the BSAC surveillance, target sample sizes were 250–280 per year for most organism collection groups and, from 2008, 500–560 for *S. aureus* and *E. coli* in bacteraemia and *S. pneumoniae* and *H. influenzae* in CA-LRTI. Targets were not always achieved so 200 is useful and reasonable as a baseline example. With a true stable resistance rate of 10% and sample size of 200 (Table S2), and ignoring other sources of variation, at least half the yearly estimates of rates would be between 8.5 and 11.5%, and at least 90% between 6.5 and 13.5%. Sampling variation alone would cause up to 10% of estimates (in this case, actually 7.5%) to be outside these limits at less than 6.5 or more than 13.5%. For a larger sample of 400

---

[1] The number N of resistant isolates in a sample of size n from a population with a proportion p of resistance has a binomial distribution, B(n,p), assuming all isolates are independent and have equal probability p of resistance. We simulated this number 100,000 times for each sample size and resistance rate using the r(binomial) function in Stata 18.0 (StataCorp, College Station, TX) to give 100,000 simulated sample estimates (N/n) of resistance prevalence.

[2] Chernick, M. R., and C. Y. Liu. 2002. The saw-toothed behavior of power versus sample size and software solutions: Single binomial proportion using exact methods. American Statistician 56: 149–155. https://doi.org/10.1198/000313002317572835.

isolates, at least 90% of estimates would be within a slightly narrower range (7.5 to 12.5%). With a smaller sample of 50 isolates/year (e.g. for a single species within an organism collection group) the range required to include at least 90% of isolates would be very much wider, at 4–18%. For this reason, we have seldom plotted annual estimates of resistance when they were based on fewer than 50 isolates/year.

The impact of such sampling variation on a 20-year series of annual estimates of resistance prevalence is illustrated in Figures S4–S6 and related notes below.

Table S2. 5th/95th and 25th/75th centiles of simulated estimates of resistance prevalence by sample size and true resistance rate

Resistance – true prevalence (%)	Sample size	% resistant: centiles of simulated estimates			
		25th	75th	5th	95th
2.0	20	0.0	5.0	0.0	10.0
2.0	50	0.0	4.0	0.0	6.0
2.0	100	1.0	3.0	0.0	5.0
2.0	200	1.5	2.5	0.5	4.0
2.0	400	1.5	2.5	1.0	3.2
2.0	800	1.6	2.4	1.2	2.9
5.0	20	0.0	10.0	0.0	15.0
5.0	50	2.0	6.0	0.0	10.0
5.0	100	3.0	6.0	2.0	9.0
5.0	200	4.0	6.0	2.5	7.5
5.0	400	4.2	5.8	3.2	6.8
5.0	800	4.5	5.5	3.8	6.2
10.0	20	5.0	15.0	0.0	20.0
10.0	50	6.0	12.0	4.0	18.0
10.0	100	8.0	12.0	5.0	15.0
10.0	200	8.5	11.5	6.5	13.5
10.0	400	9.0	11.0	7.5	12.5
10.0	800	9.2	10.8	8.2	11.8
20.0	20	15.0	25.0	5.0	35.0
20.0	50	16.0	24.0	12.0	30.0
20.0	100	17.0	23.0	14.0	27.0
20.0	200	18.0	22.0	15.5	24.5
20.0	400	18.8	21.2	16.8	23.2
20.0	800	19.0	21.0	17.8	22.4

Distributions simulated at each sample size by 100,000 replicate draws from binomial distribution B(n,p) with p (probability) equal to true prevalence and n (number) equal to sample size.

Figure S2. 20% resistance: centiles of simulated estimates for samples of [A] 20–900 isolates; [B] 100–500 isolates.

A

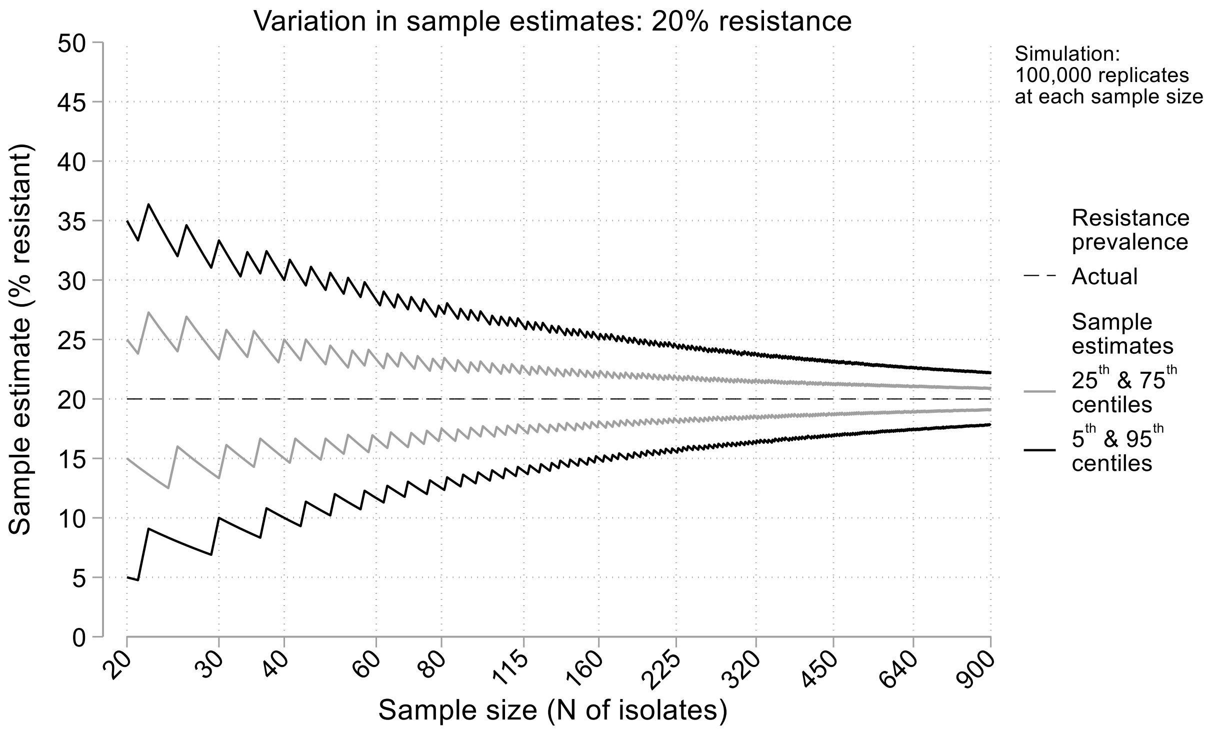

B

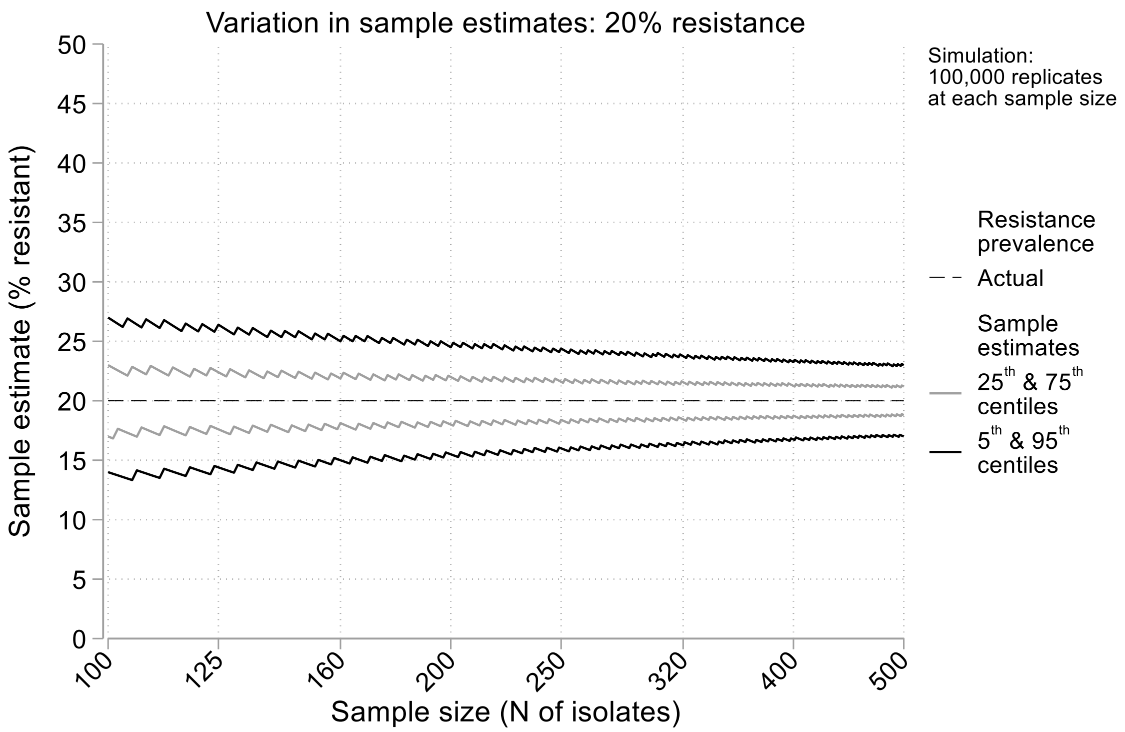

Note log scale for sample size.

Figure S3. 10% resistance: centiles of simulated estimates for samples of [A] 20–900 isolates; [B] 100–500 isolates.

A

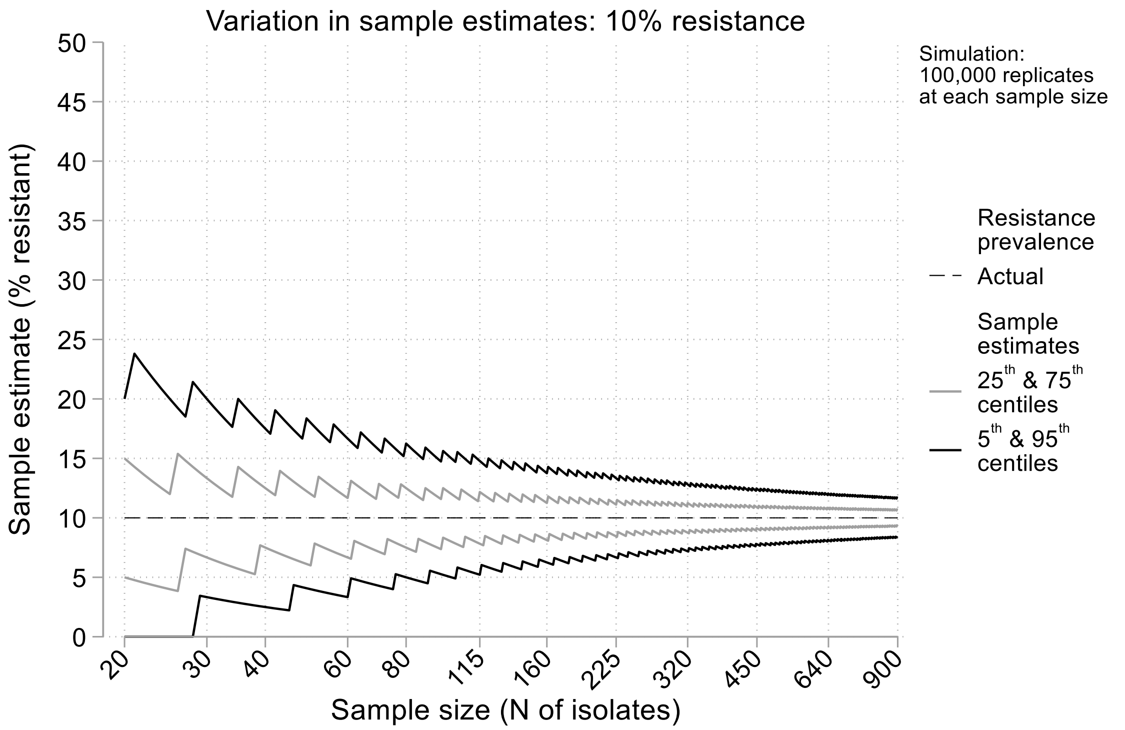

B

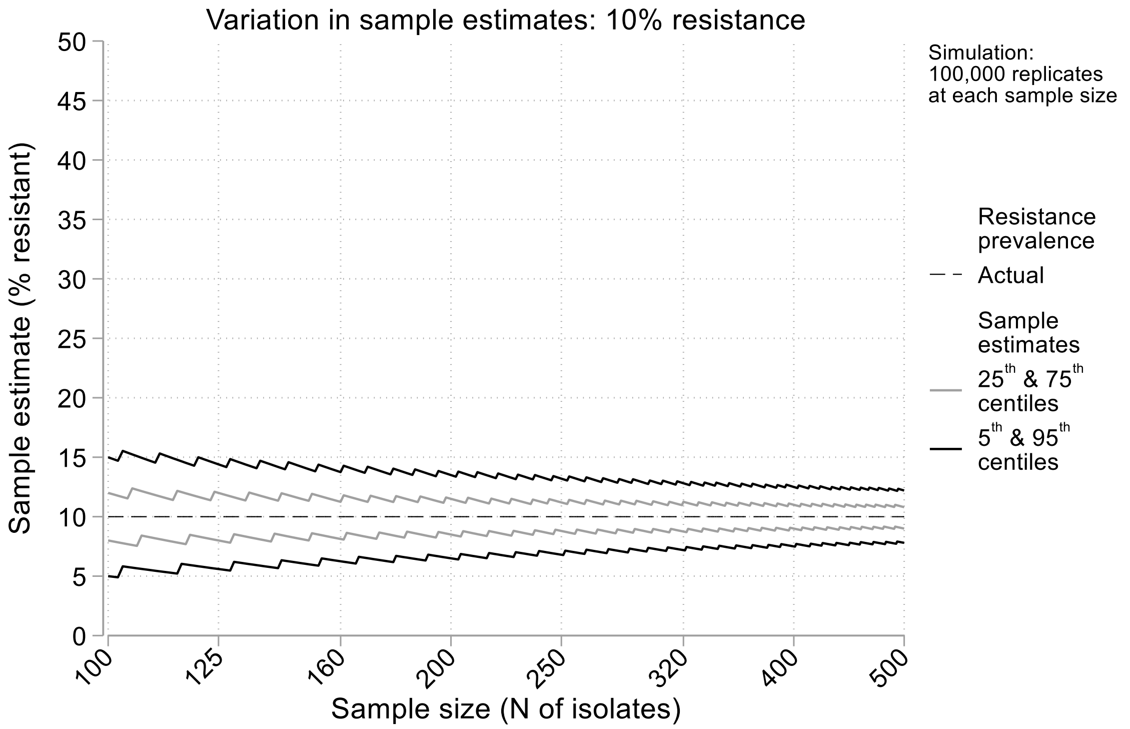

Note log scale for sample size.

Figure S4. 5% resistance: centiles of simulated estimates for samples of [A] 20–900 isolates; [B] 100–500 isolates.

A

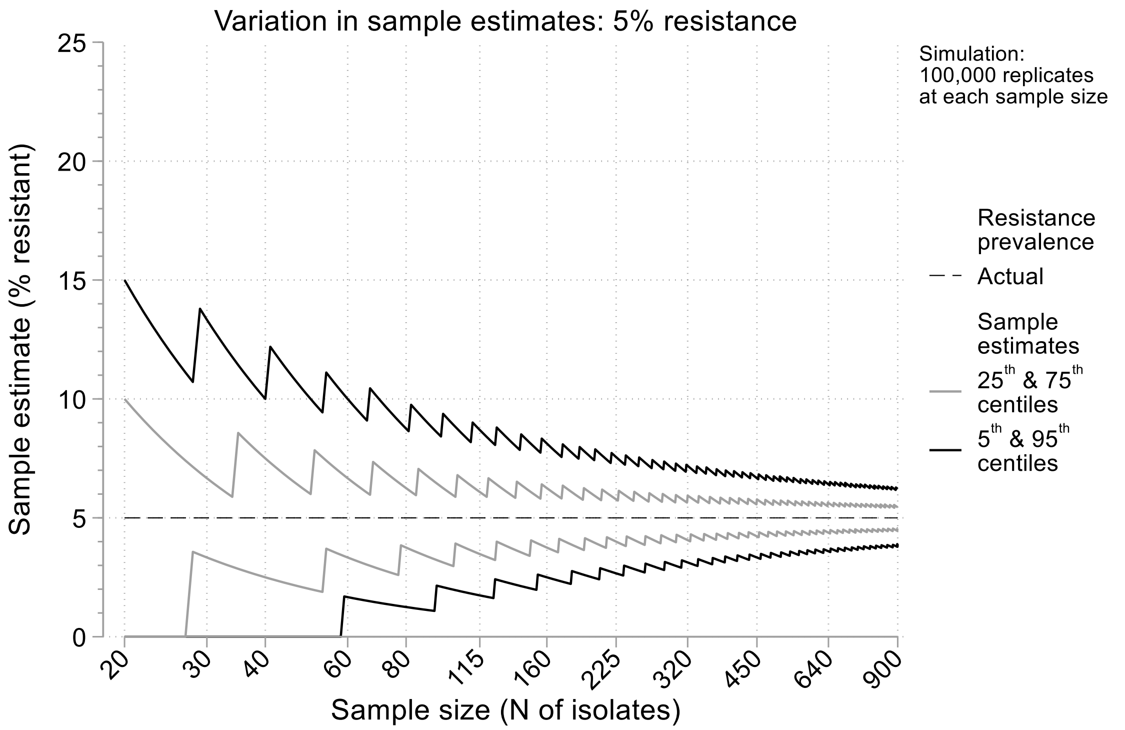

B

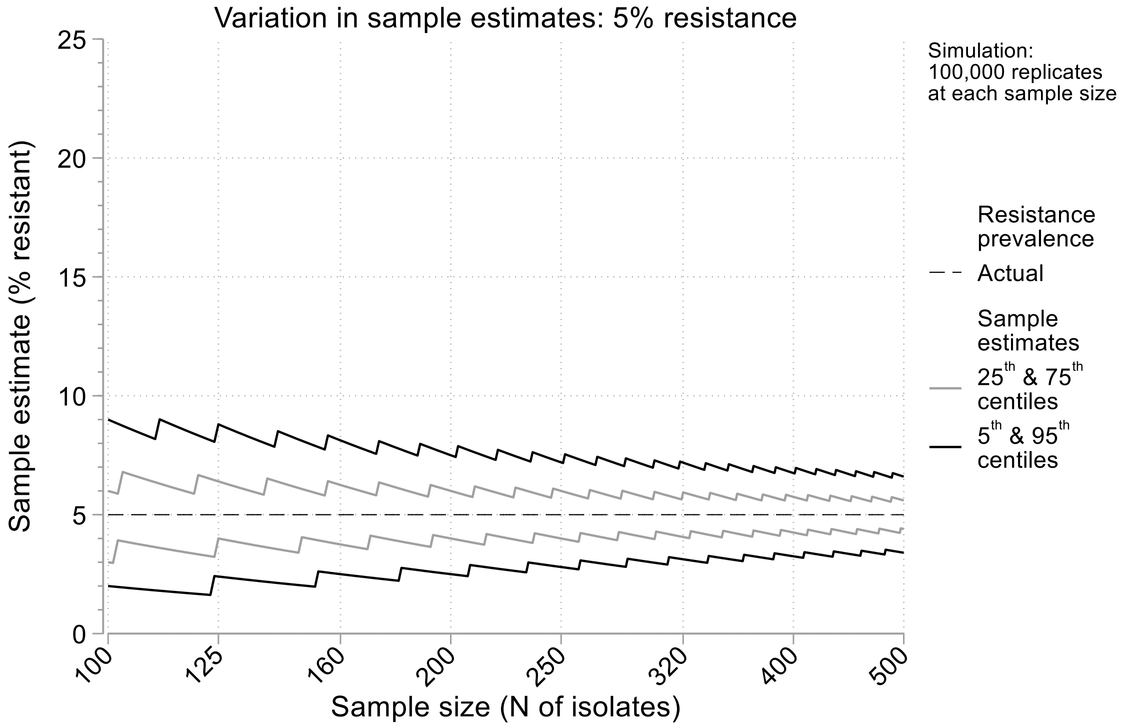

Note log scale for sample size.

Figure S5. 2% resistance: centiles of simulated estimates for samples of [A] 20–900 isolates; [B] 100–500 isolates.

A

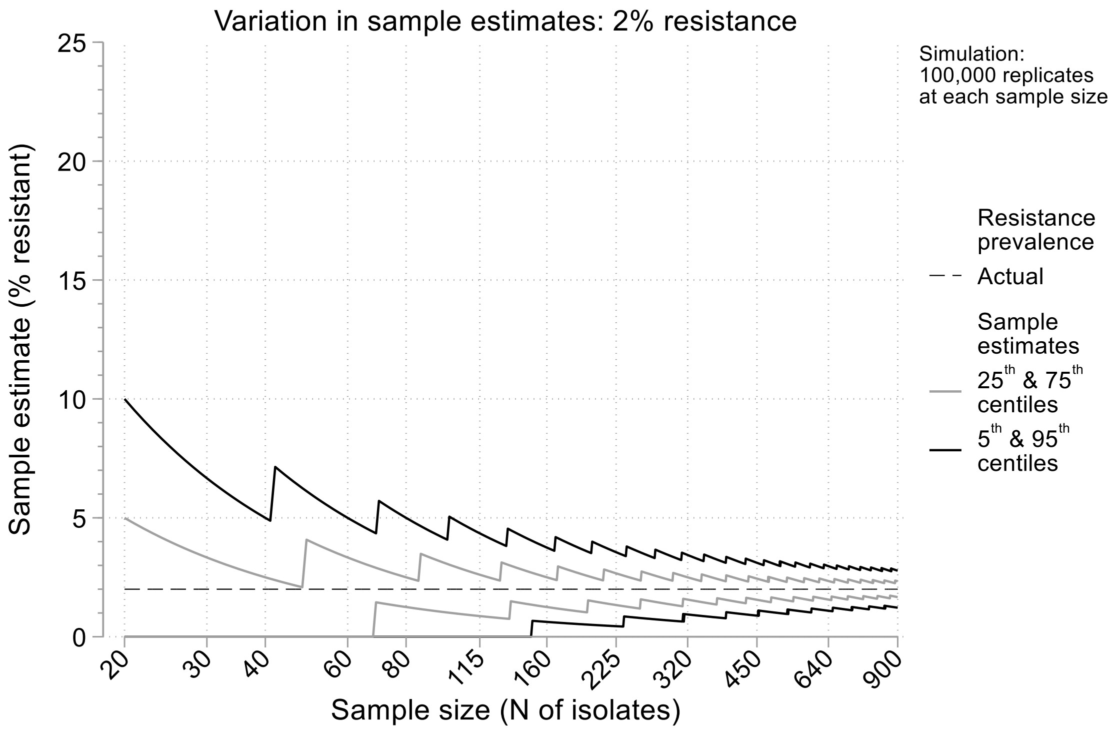

B

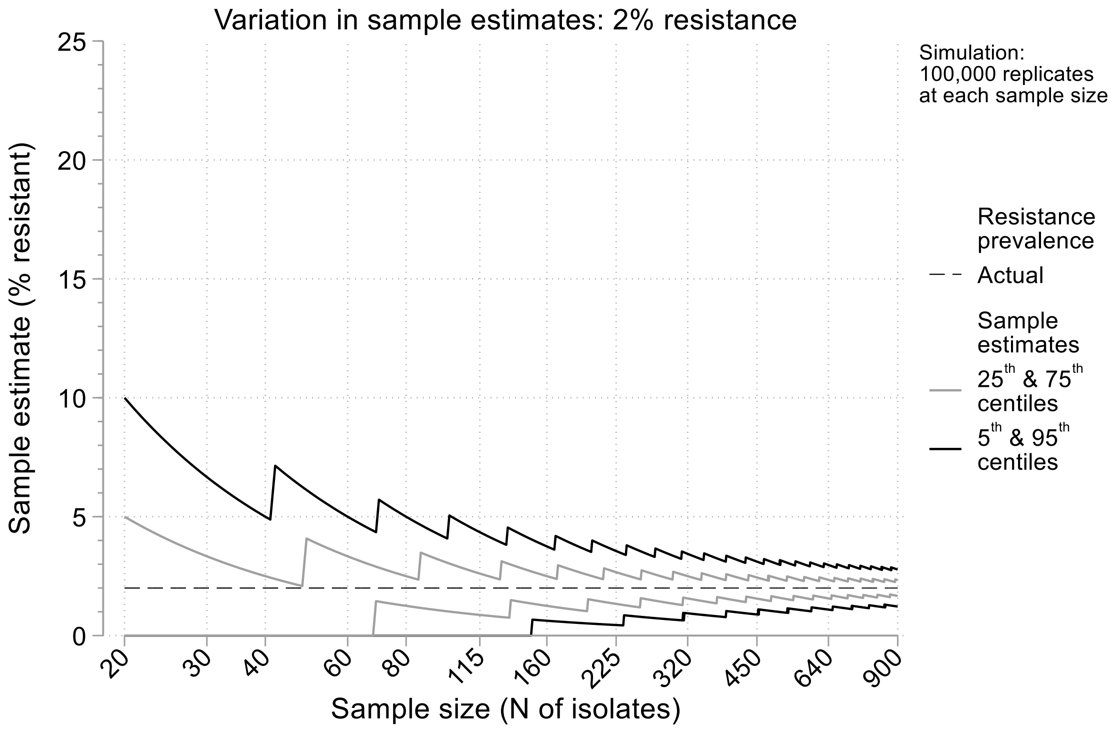

Impact of variation in 20-year surveillance series, illustrated by simulation

A long series of annual resistance estimates gives many opportunities for individual values to fall outside the 5th to 95th centile range described above, each estimate having a chance of up to 10%. Over a 20-year series, it would be commonplace (up to 75% chance) to find 1–3 years with estimates outside these limits (at <6.5% or >13.5%, if true resistance rate was 10%) and less usual to see more than 3 (up to 13% chance) or none (at least 12% chance).

Factors not accounted for by this modelling, such as outbreaks, differences in resistance by centre, centre turnover and experimental (laboratory) variation over time will all add to the variability.

Figure S6. Six random examples of variation over simulated 20-year series for stable 10% resistance, sample size 200

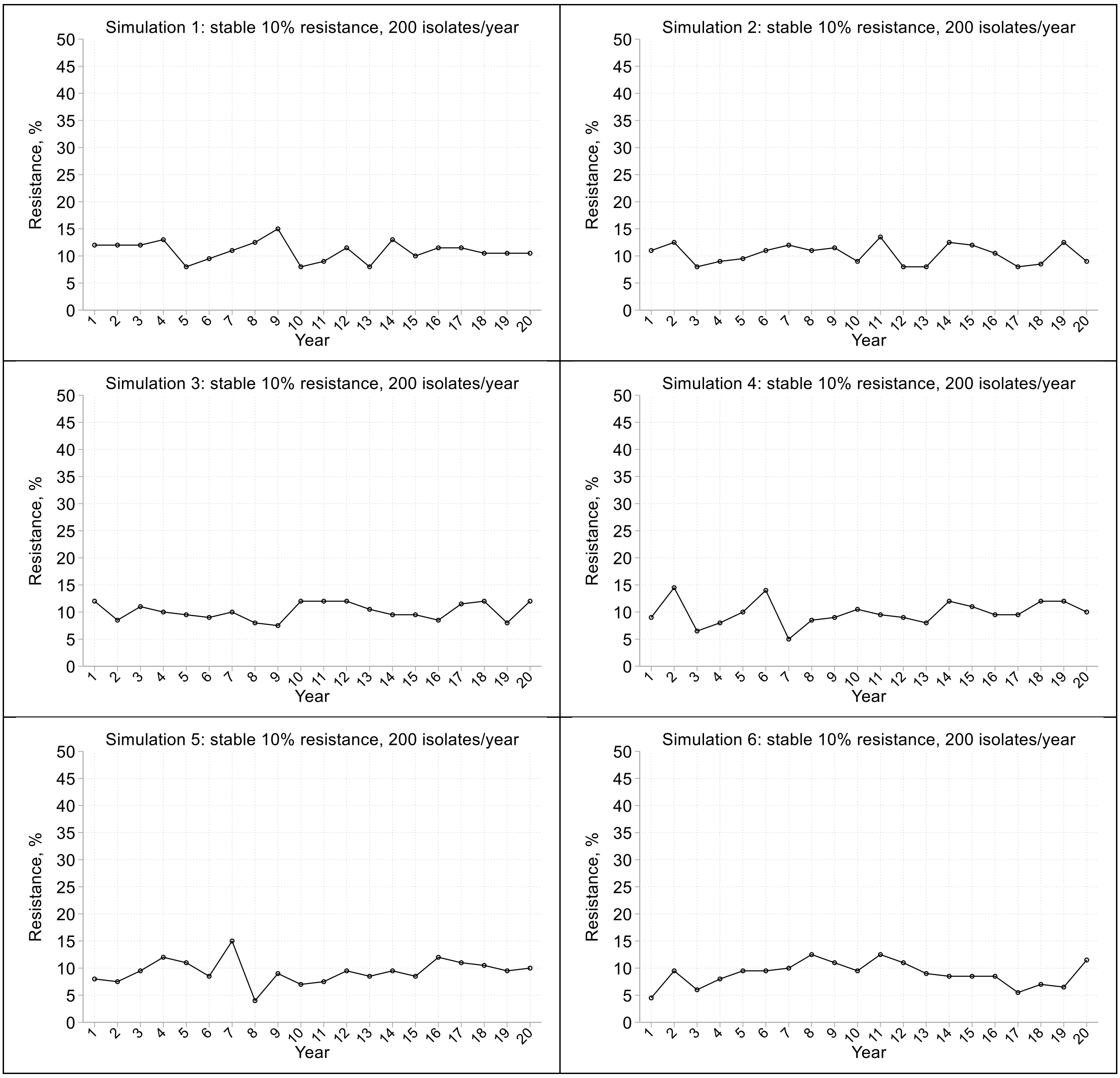

Figure S7. Comparison by sample size: six superimposed simulated examples of 20-year series for stable 10% resistance

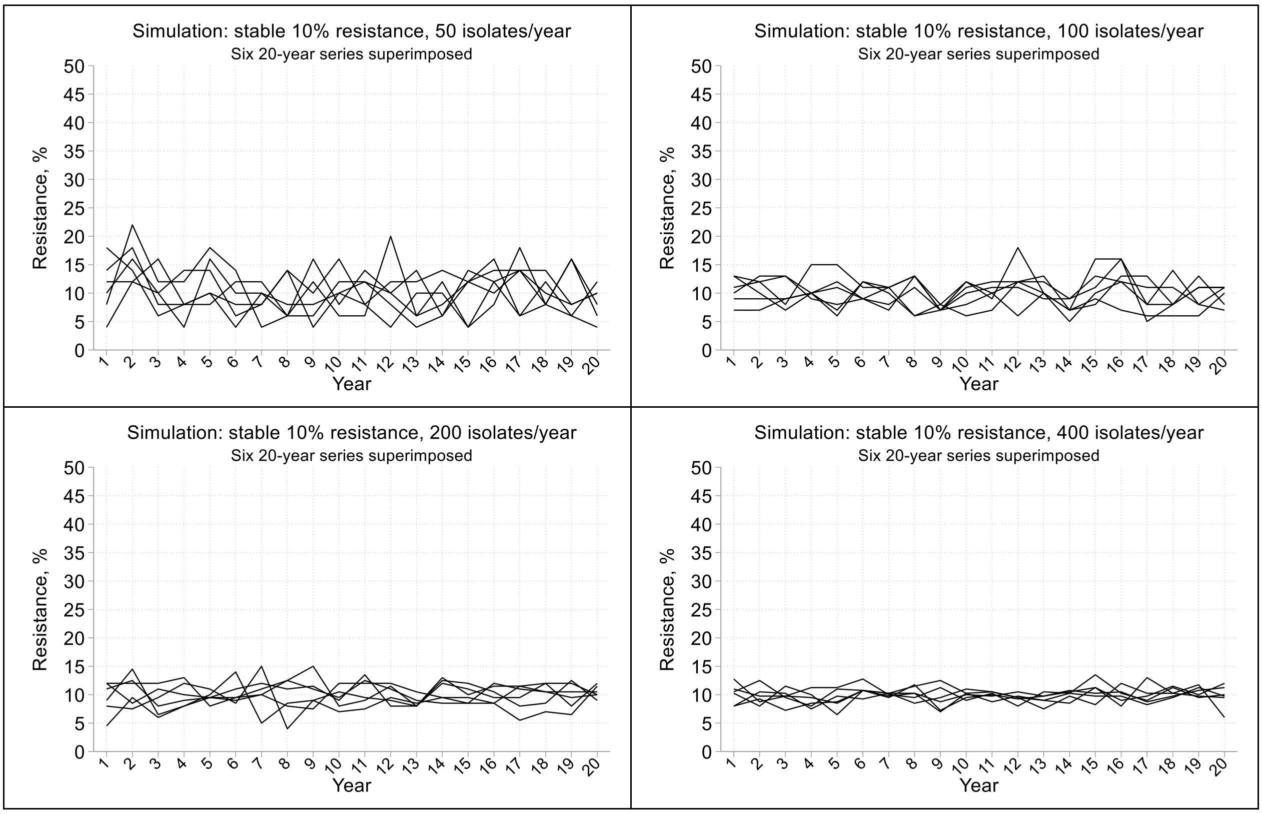

Figure S8. Comparison by true resistance rate: six superimposed simulated examples of 20-year series with 200 isolates/year

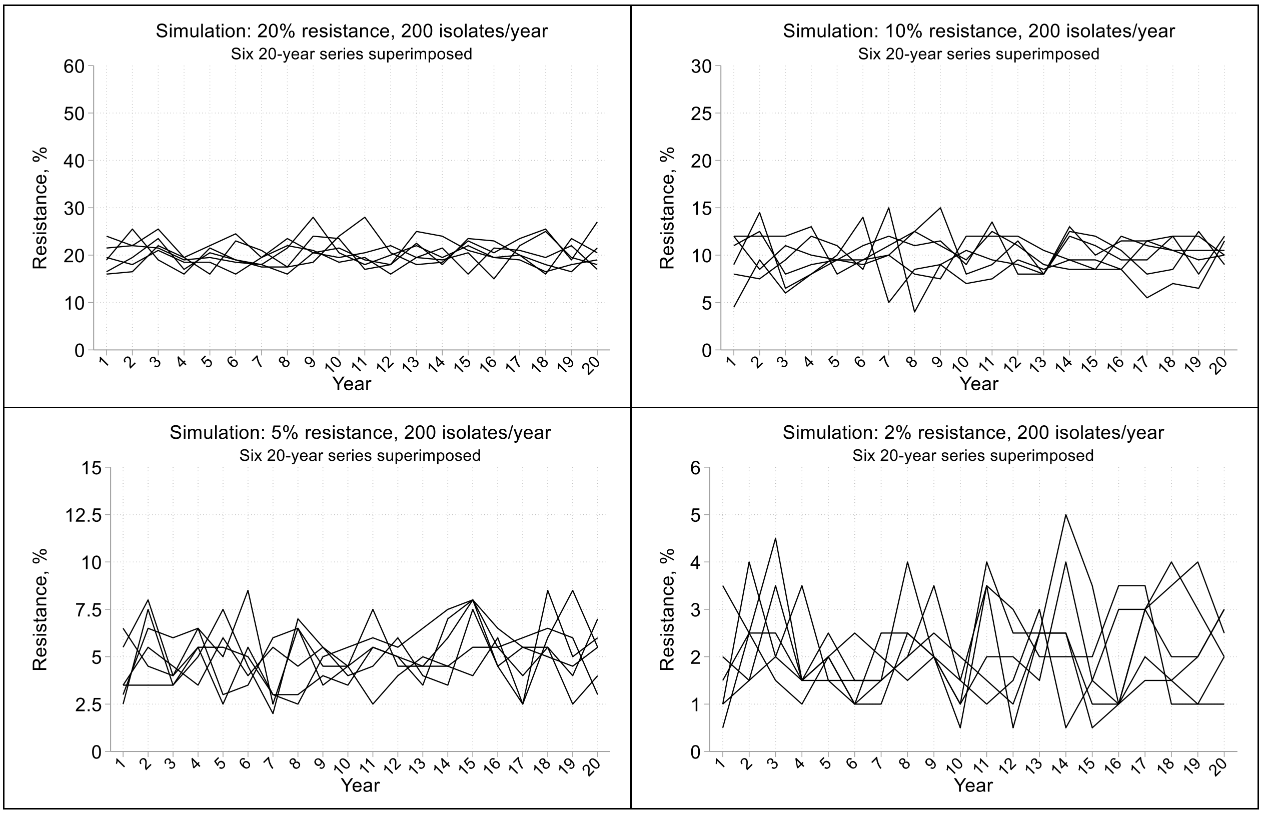

The y-axes (resistance scales) for these four plots are comparable: the 'true' underlying resistance rate is at one-third height in all cases, so the visual impression is of the variability relative to the true resistance rate.

Visual impact of graph scale in 20-year surveillance series

Figure S9. Four simulated examples of 20-year series for stable 10% resistance presented on two scales: y-axis 0–20% or 0–50%

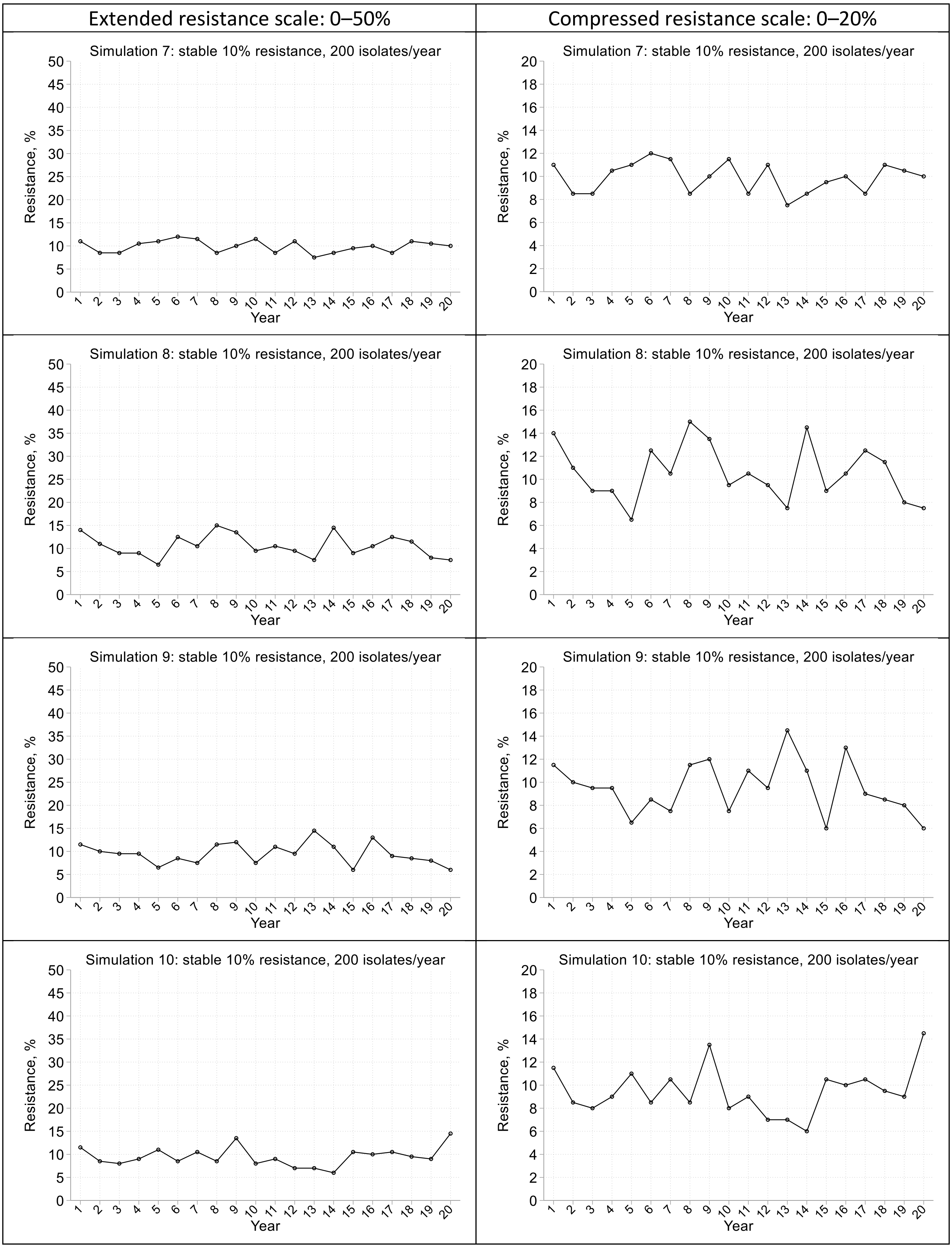

When measurements are very accurate, it is good practice to ‘zoom in’ and plot them on a scale that spreads the points out, but this is unhelpful when estimates are subject to substantial random variation, as here, as it tends to exaggerate the visual impression of (spurious) trends.
